# Nonpromoter methylation of the CDKN2A gene with active transcription is associated with improved locoregional control in laryngeal squamous cell carcinoma

**DOI:** 10.1002/cam4.961

**Published:** 2017-01-19

**Authors:** Miriam M. Ben‐Dayan, Thomas J. Ow, Thomas J. Belbin, Joshua Wetzler, Richard V. Smith, Geoffrey Childs, Brenda Diergaarde, D. Neil Hayes, Jennifer R. Grandis, Michael B. Prystowsky, Nicolas F. Schlecht

**Affiliations:** ^1^ Department of PathologyAlbert Einstein College of MedicineMontefiore Medical CenterBronxNew York; ^2^ Department of Otorhinolaryngology‐Head and Neck SurgeryAlbert Einstein College of MedicineMontefiore Medical CenterBronxNew York; ^3^ Dicipline of OncologyFaculty of MedicineMemorial University of NewfoundlandSt. John'sNewfoundland; ^4^ Department of EpidemiologyUniversity of Pittsburgh Cancer InstitutePittsburghPennsylvania; ^5^ Department of Otolaryngology/Head and Neck Cancer SurgeryUniversity of North CarolinaChapel HillNorth Carolina; ^6^ Departments of Otolaryngology Head and Neck SurgeryUniversity of CaliforniaSan FranciscoCalifornia; ^7^ Department of Clinical and Translational Science InstituteUniversity of CaliforniaSan FranciscoCalifornia; ^8^ Departments of Epidemiology & Population Health and MedicineAlbert Einstein College of MedicineBronxNew York

**Keywords:** CDKN2A, gene transcription, larynx cancer, methylation, p14(ARF), p16(INK4a), tumor biomarkers

## Abstract

We previously reported a novel association between CDKN2A nonpromoter methylation and transcription (ARF/INK4a) in human papillomavirus associated oropharyngeal tumors. In this study we assessed whether nonpromoter CDKN2A methylation in laryngeal squamous cell carcinomas (LXSCC) conferred a similar association with transcription that predicted patient outcome. We compared DNA methylation and ARF/INK4a RNA expression levels for the CDKN2A locus using the Illumina HumanMethylation27 beadchip and RT‐PCR in 43 LXSCC tumor samples collected from a prospective study of head and neck cancer patients treated at Montefiore Medical Center (MMC). Validation was performed using RNAseq data on 111 LXSCC tumor samples from the Cancer Genome Atlas (TCGA). The clinical relevance of combined nonpromoter CDKN2A methylation and transcription was assessed by multivariate Cox regression for locoregional recurrence on a subset of 69 LXSCC patients with complete clinicopathologic data from the MMC and TCGA cohorts. We found evidence of CDKN2A nonpromoter hypermethylation in a third of LXSCC from our MMC cohort, which was significantly associated with increased ARF and INK4a RNA expression (Wilcoxon rank‐sum, *P* = 0.007 and 0.003, respectively). A similar association was confirmed in TCGA samples (Wilcoxon rank‐sum test *P* < 0.0001 for ARF and INK4a). Patients with CDKN2A hypermethylation or high ARF/INK4a expression were significantly less likely to develop a locoregional recurrence compared to those with neither of the features, independent of other clinicopatholgic risk factors (adjusted hazard ratio=0.21, 95% confidence interval:0.05–0.81). These results support the conclusion that CDKN2A nonpromoter methylation is associated with increased ARF and INK4a RNA expression, and improved locoregional control in LXSCC.

## Introduction

There are an estimated 13,430 new cases of laryngeal squamous cell carcinomas (LXSCC) diagnosed each year in the U.S 1. Advanced stage LXSCC is treated with radiation or chemoradiation, with current strategies favoring organ‐sparing approaches to reduce comorbidity 2. Despite well‐established multimodality protocols, locoregional failure remains at 20–30% 3. Whereas the detection of human papillomavirus (HPV) is an important predictor of clinical outcome in oropharyngeal squamous cell carcinomas (OPSCC) 4, HPV is much less prevalent in LXSCC 5, and there are no molecular or genomic biomarkers to guide treatment selection for LXSCC patients.

Recently, our group identified a novel association between methylation in the nonpromoter region of CDKN2A and increased p16(INK4a) expression in HPV‐positive OPSCC 6. The CDKN2A gene, located at chromosome 9p21, encodes two distinct tumor suppressor proteins, INK4a and ARF. While the transcripts overlap, they each incorporate a unique first exon and different reading frames 7. Furthermore, whereas both CDKN2A proteins are known to induce cell cycle arrest and are commonly disrupted during tumorigenesis 7, p16(INK4a) expression is not common in LXSCC and is not predictive of clinical outcome 8. However, the expression of ARF is being assessed as a therapeutic alternative in HPV‐negative head and neck cancer 9. We hypothesized that nonpromoter hypermethylation of CDKN2A and associated transcription may occur in a subset of less aggressive LXSCC.

To test this hypothesis, we examined patients with LXSCC enrolled in an ongoing research program at a large urban health center to measure CDKN2A nonpromoter methylation and transcription in tumors. We assessed whether increased methylation and expression of the CDKN2A region was associated with locoregional control in these patients. We validated our findings in an independent sample of LXSCC tumors with genetic, epigenetic, and transcriptomic data from the Cancer Genome Atlas (TCGA; http://cancergenome.nih.gov/) 10.

## Methods

### Patient tissue samples

Initial analyses were based on 43 histologically confirmed LXSCC tumors obtained by biopsy or surgical resection from patients undergoing treatment at MMC in Bronx, NY between 2002 and 2010. All patients provided written consent for participation in this study under a protocol approved by the Institutional Review Board at MMC. All tumors were assessed histologically to ensure a majority of tumor cells in samples. Tumor samples were snap‐frozen in liquid nitrogen within 30 min and kept at −80°C until DNA and RNA analysis. This cohort served as our discovery sample set.

All tumor samples were tested for HPV‐16 RNA using a Real‐Time Reverse Transcription‐Polymerase Chain Reaction (qRT‐PCR) assay described previously 11. Tumors with high E6 and/or E7 RNA expression (∆CT ≥100‐fold compared to GAPDH) were considered HPV‐positive. Presence of p16(INK4a) protein was also assayed using a clinically available immunohistochemistry (IHC) antibody (BD Biosciences, San Jose, CA; Cat. no. 551154) 8. Briefly, p16 staining was done for formalin‐fixed and paraffin‐embedded tumor section. Tumors were considered p16‐positive if >50% of the tumor cells exhibited strong nuclear staining.

### Methylation analyses

The protocol for measuring DNA methylation using microarrays was as described previously 11. For methylation analysis, HumanMethylation27 beadchip (Illumina, San Diego, CA) beta values were transformed to normalized M‐values using an established protocol 12. The previously described nonpromoter region included one CpG within exon 3 (cg11653709) and three CpGs within the preceding intron (cg07752420, cg09099744, and cg10895543) 6. In addition to the nonpromoter region, we assessed methylation levels for 3 CpG loci within the ARF promoter of CDKN2A (cg00718440, cg03079681, and cg26673943). We also tested adjacent normal tissues from 48 LXSCC patients but found consistently low levels of methylation overall in all samples (data not shown).

### RNA expression analyses

Expression levels for ARF and INK4a were quantified from total RNA (100 ng) of 29 LXSCC tumors using real‐time reverse transcriptase‐PCR (qRT‐PCR) Taqman^®^ probes and the TaqMan RNA‐to‐Ct^™^ 1‐Step Kit (Applied Biosystems, Waltham, MA). Resulting cycle threshold (Ct) values were normalized to GAPDH and subsequently converted to relative expression (2^−ΔΔCT^) values.

As an additional platform, whole‐genome expression microarray data from a prior analysis of head and neck tumors were also assessed 11 and 21 of the LXSCC samples used for the methylation analysis were used in this analysis. TRIzol (Ambion, Austin, TX) extracted RNA (500 ng) for each tumor sample was amplified and biotin labeled using the Illumina TotalPrep RNA Amplification Kit (Ambion, Austin, TX). Messenger RNA expression levels were subsequently analyzed by RNA hybridization to the Illumina HumanHT‐12‐v3 Expression BeadChip (Illumina, San Diego, CA). Microarray probes for the CDKN2A gene were identified using the RefSeq database release 17 and UniGene build 188. RNA expression levels were assessed by a probe targeting ARF and a combined CDKN2A probe. In addition, a probe corresponding to the INK4b gene in the adjacent CDKN2B region was also identified and used to measure corresponding expression of that transcript.

### *TCGA* data and analyses

Publically available genomic data from The Cancer Genome Atlas (TCGA) head and neck cancer project were utilized as a validation set for these studies 10. CDKN2A methylation, RNA expression, and patient clinical data were mined for 111 LXSCC tumor samples from the TCGA database. Per TCGA protocol, only samples with >60% tumor cells were included for analysis. One CpG (cg12840719) located within the same downstream nonpromoter region of the CDKN2A gene was captured by the HumanMethylation 450K beadchip (Illumina, San Diego, CA) array used by TCGA. We previously showed this region to exhibit consistent methylation patterns across neighboring CpGs 6.

Other data extracted from TCGA included RNA expression and genetic mutation events corresponding to the CDKN2A gene. Reads Per Kilobase per Million mapped reads (RPKM) for ARF, INK4a, Cylcin A, Cyclin E, and INK4b from TCGA were used to measure gene expression levels generated by RNAseq 10. HPV expression status was determined using E6 and E7 RNAseq data 10. In addition, level 3 copy number variation (CNV) data for 9p21 and the CDKN2A locus, and level 3 somatic mutation data for CDKN2A and TP53 were mined for all TCGA LXSCC samples 10.

### Statistical analyses

For efficiency and purposes of statistical validation, the MMC discovery cohort samples were divided into two groups based on CDKN2A methylation levels (hypo‐ and hyper‐methylated) using Euclidean distance hierarchical clustering with complete linkage using all 4 CpG loci. The corresponding methylation heatmap was visualized using the supraHex v1.8.0 R package 13. To allow for statistical comparison, grouping of the TCGA samples was based on the distribution of M‐values for the single downstream CDKN2A CpG (cg12840719) assayed by the HumanMethylation 450K beadchip assuming a cutoff for hypermethylation based on the same percentile threshold of 32% observed in our discovery sample of LXSCC tumors from MMC (M = −0.048). CpG loci located at the promoters of ARF and INK4a transcripts were also assessed from TCGA data.

With respect to the expression data, MMC samples were grouped based on median RNA values for each transcript excluding LXSCC tumors with no detectable RNA, with relative expression thresholds of 49 (ΔΔCT) for ARF and 166 (ΔΔCT) for INK4a. The cut‐off selection for ARF and INK4a expression in TCGA samples was similarly based on median RNA levels excluding tumors with no detectable RNA (with a corresponding RPKM of 171 for ARF and 35 for INK4a). This was done to differentiate between tumors with high and low RNA expression.

Wilcoxon rank sum tests were used to determine the statistical significance of observed differences in CDKN2A transcript levels between tumors with hypo‐ or hypermethylated CDKN2A. Box‐and‐Whisker plots were generated using the ggplot2 v2.0.0 R package 14. We used contingency tables to assess the associations between CDKN2A locus methylation/expression status (i.e., comparing tumors with high vs. low CDKN2A methylation/expression) and clinicopathologic factors at diagnosis, and tested using Chi‐square or Fisher Exact test statistics, where appropriate. All tests were two‐sided.

In addition to the 29 patients followed at MMC, information on clinical outcome was ascertained for 69 LXSCC patients originating from three collaborating TCGA tissue collection sites: the University of Pittsburgh (*n* = 23), Vanderbilt University (*n* = 9), and the University of North Carolina (UNC) at Chapel Hill (*n* = 8). Response to treatment was based on locoregional recurrence, defined as a recurrence of cancer at the primary site or regional lymph nodes. The association between CDKN2A locus methylation/expression and time to locoregional recurrence was assessed using Kaplan–Meier survival analyses, and strata were compared using Log‐rank test statistics. Multivariable Cox regression models were built, adjusting a priori for gender, age (<60 vs. ≥60 years) and regional lymph node metastases 15 [node negative (N0) vs. regional lymph node metastasis (N+)]. Other clinicopathologic variables assessed included treatment (coded as unimodality treatment [radiation or surgery], surgery plus postoperative adjuvant treatment [radiation or chemoradiation], and primary chemoradiation), overall stage 16 and tumor size 15 (I/II vs. III/IV), smoking status (current vs. former smoker), and HPV status. All models were stratified by sample cohort (MMC vs. TCGA) in order to adjust for possible bias associated with tissue acquisition protocols for the different studies and methylation platforms. A parsimonious model was derived using backward elimination for variables with *P* < 0.05. Effect modification (e.g., with clinical factors and study site) was tested for by including cross‐product terms into the model. The potential for confounding was also assessed using a change in point criterion 17. Proportional hazards assumptions were tested using log‐log plots, observed versus expected plots, and goodness‐of‐fit tests. Kaplan–Meier plots were generated using the Survival v2.38.3 R package^18^, and multivariable Cox regression analyses were performed using Stata v.14 (StataCorp, College Station, TX).

## Results

### A subset of LXSCC tumors exhibit CDKN2A nonpromoter methylation

**Figure 1:**
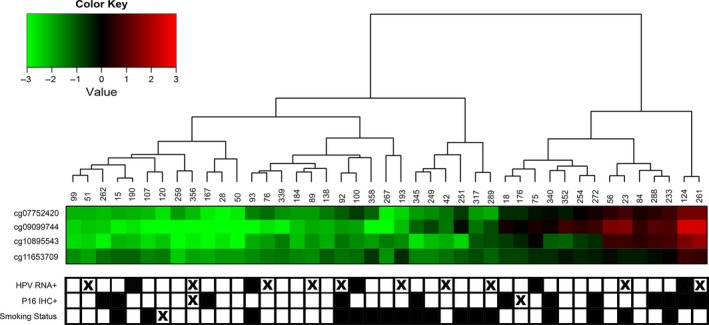
Cluster analysis of laryngeal tumor samples based on CDKN2A nonpromoter methylation. The M‐values of 4 CpGs from the downstream nonpromoter region of CDKN2A were heirarchically clustered for 43 primary LXSCC tumors. Each leaf in the dendrogram represents a sample, and each row represents an individual CpG loci. Two main clusters were identified based on the dendrogram above the heatmap. Samples in the left, mostly green cluster, were categorized as the hypomethylated group (*n* = 29), and the samples in the right, mostly red cluster, were categorized as the hypermethylated group (*n* = 14). HPV RNA, p16(INK4a) protein expression (by IHC), and smoking status for each sample are shown below the heatmap. White squares indicate HPV RNA negative, p16(INK4a) protein negative and noncurrent smokers, respectively. Black squares indicate HPV RNA positive, p16(INK4a) protein positive and current smokers, respectively. Missing data are denoted by an “X”. A color key matching color with corresponding M‐value is shown in the upper left corner. HPV, human papillomavirus; IHC, immunohistochemistry.

We first set out to identify CDKN2A nonpromoter methylation differences between LXSCC tumors. Using hierarchical clustering, 43 primary tumor samples from patients treated at MMC were clustered by the methylation array data for four CpGs within the downstream nonpromoter region of CDKN2A (Fig. 1). Two clusters of tumors were identified with approximately a 2‐to‐1 ratio with hypo‐ and hypermethylation.

By clustering the methylation values for the four nonpromoter CpGs, we were able to compare LXSCC tumors with and without CDKN2A methylation. The clinical characteristics for the MMC patients included in our analyses are summarized in Table S1. Hypermethylated LXSCC tumors were more likely to have p16(INK4a) protein expression as measured by IHC compared to hypomethylated tumors (Fisher's Exact test *P* = 0.029). In contrast, the prevalence of HPV was much lower and showed no significant association with CDKN2A methylation (*P* = 1.0). No significant differences were observed with respect to other clinicopathologic factors between the hyper‐ and hypomethylated groups.

### CDKN2A nonpromoter methylation is associated with ARF and INK4a RNA expression

**Figure 2:**
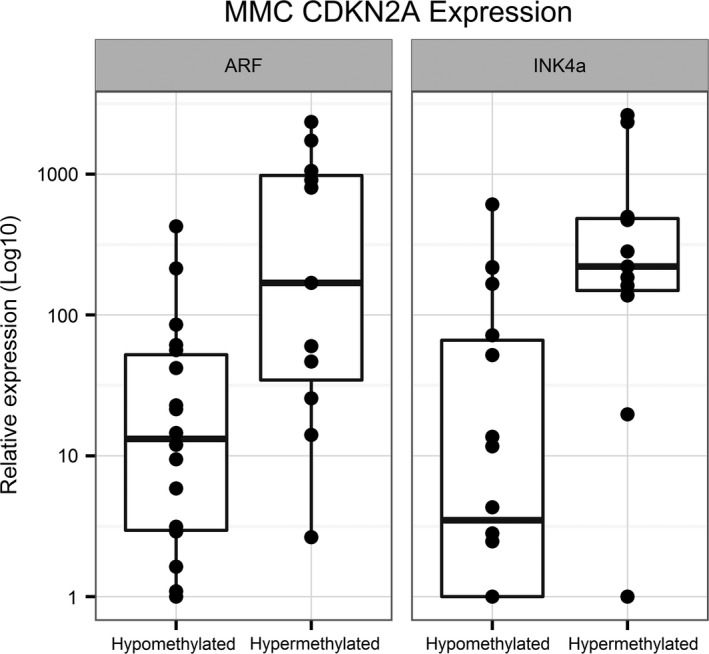
Overexpression of CDKN2A transcripts in laryngeal tumor samples with CDKN2A nonpromoter methylation. Relative mRNA expression values obtained by qRT‐PCR for ARF and INK4a were plotted by tumor methylation status on a Log10 scale. Eighteen hypomethylated LXSCC tumors were compared to 11 hypermethylated tumors for each transcript. Increased levels of ARF (Wilcoxon rank‐sum, *P* = 0.0068) and INK4a (*P* = 0.003) were seen in the hypermethylated groups.

As we did previously for OPSCC, we also examined the relationship between CDKN2A nonpromoter methylation and RNA expression in LXSCC. We measured RNA levels for INK4a and ARF by qRT‐PCR in 29 LXSCC tumors. The clinical characteristics for the patients with qRT‐PCR data are summarized in Table S2. Increased levels of both ARF and INK4a were found in tumors with CDKN2A hypermethylation compared to those without (Wilcoxon rank‐sum test *P* = 0.007 and 0.003, respectively; Fig. 2). RNA levels for these transcripts were also measured using the Illumina expression array as an alternative platform for validation. Of the 33 LXSCC tumors analyzed using the Illumina expression array, 26 were also assayed by qRT‐PCR. Consistent with the qRT‐PCR results, ARF and CDKN2A RNA levels were increased in tumors with nonpromoter CDKN2A hypermethylation (*P* < 0.0001 for both probes; Fig. S1).

ARF and INK4a RNA levels were also plotted against the methylation M‐values for each of the four nonpromoter CpG loci tested on the Illumina methylation array revealing significant positive correlations with ARF and INK4a transcription (Figs S2 and S3). Methylation of CpG loci within the promoter region of ARF were also measured, but these showed no evidence of methylation and no correlation with corresponding RNA expression (data not shown). Together, these data indicate that increased ARF and INK4a RNA levels are associated with CDKN2A nonpromoter hypermethylation.

### TCGA LXSCC tumors validate our methylation and expression results

To validate our results from the MMC cohort, we analyzed CDKN2A nonpromoter methylation and RNA levels for 111 LXSCC tumors available in the TCGA database. Table S3 summarizes the clinical characteristics for the patients in TCGA. Unlike the Illumina HumanMethylation27 beadchip array used in our MMC study, which assayed four downstream nonpromoter CDKN2A CpG loci, collection of DNA methylation data for TCGA samples utilized the Illumina HumanMethylation 450K beadchip platform, which assayed only one novel CpG within the region of interest. For consistency, we selected an M‐value cutoff based on the distribution in methylation observed for the MMC cohort (see Methods for cutoff); ARF and INK4a RNA levels were plotted against CpG M‐values to demonstrate the correlation between hypermethylation and increased RNA levels for CDKN2A expression (Fig. S4).

**Figure 3:**
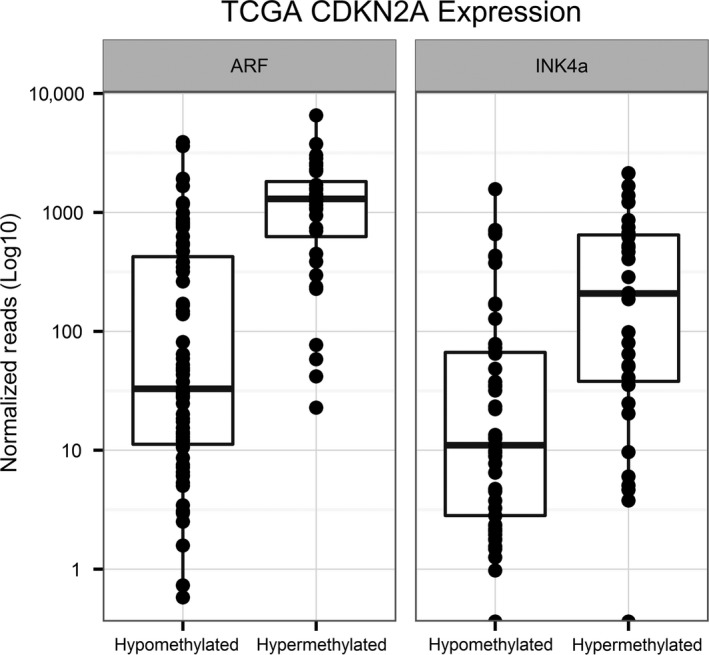
Overexpression of CDKN2A transcripts in hypermethylated TCGA laryngeal tumors. RNAseq expression values for ARF and INK4a were plotted for 111 primary LXSCC tumors from the TCGA cohort. Seventy‐five hypomethylated tumors were compared to 36 hypermethylated tumors with the expression levels plotted on a Log10 scale. We observed an increase in ARF (Wilcoxon rank‐sum, *P* < 0.0001) and INK4a (*P* < 0.0001) expression for the hypermethylated groups.

We compared the CDKN2A RNA levels obtained by RNAseq for 75 samples that were classified as hypomethylated and 36 samples that were hypermethylated (Fig. 3). The hypermethylated LXSCC tumors exhibited an increase in both ARF and INK4a RNA compared to hypomethylated tumors (Wilcoxon rank‐sum test *P* < 0.0001 for both transcripts), confirming the results seen in our MMC cohort. Alternative cutoffs were evaluated, but moving the methylation threshold by one standard deviation (equivalent to −1.7 M‐values) did not affect our results. In addition, we were able to assess the methylation levels for the promoter regions of both CDKN2A transcripts, which were uniformly unmethylated, ruling out the possibility that the lack of CDKN2A RNA in hypomethylated tumors was due to promoter methylation. As such, data from TCGA LXSCC tumors validated our MMC findings that CDKN2A nonpromoter methylation was associated with ARF and INK4a RNA expression.

### Tumors with high CDKN2A expression exhibit CDKN2A and/or TP53 mutations

**Figure 4:**
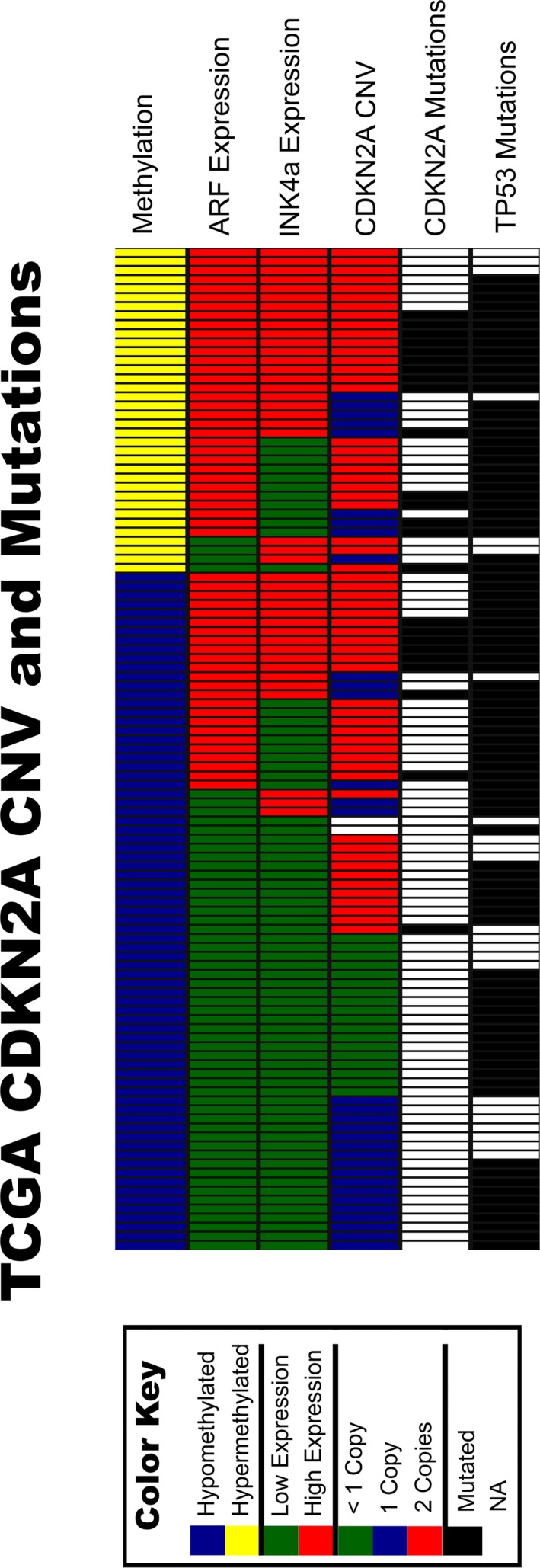
Copy number variation and mutation data for TCGA LXSCC cohort. All 111 LXSCC from the TCGA cohort were sorted based on CDKN2A methylation, ARF expression, INK4a expression, CDKN2A copy number variation (CNV), CDKN2A mutation, and TP53 mutation status. Each column represents a single sample. A color key indicating CDKN2A methylation status (hypo vs. hyper), CDKN2A RNA expression status (low vs. high), CDKN2A CNV, and mutations status (CDKN2A and TP53) is shown on the left.

Given that the CDKN2A locus encodes two tumor suppressors, and that both transcripts are elevated in LXSCC tumors, we wanted to examine the presence of genetic variations across this locus. Data available from TCGA allowed for the assessment of genetic alterations, including 9p21 CNV, CDKN2A mutation status, and p53 mutation status (Fig. 4). As expected, tumors with homozygous deletions for CDKN2A were only observed in the group with low CDKN2A RNA, and in the majority of the hypomethylated LXSCC. However, 58% of hypomethylated tumors with low CDKN2A RNA expression had at least one copy of CDKN2A. Moreover, at least one copy was retained for all tumors with high levels of ARF and INK4a RNA, and 75% of the hypermethylated tumors retained both copies. Many tumors with high levels of CDKN2A RNA also exhibited mutations within the CDKN2A (22%) and/or TP53 (89%) loci. All the CDKN2A mutations were seen within the first exon of the INK4a transcript (exon 1*α*) and the shared exon 2, and there were no mutations within the first exon of the ARF transcript (exon 1*β*). These data suggest that despite high levels of ARF and INK4a RNA, mutations within the CDKN2A and TP53 loci may be a hindrance toward fully functional tumor suppressors.

### RNA levels of E2F targets remain unaffected by CDKN2A nonpromoter methylation

Since INK4a acts as an inhibitor of E2F transcription factors, we also examined the RNA expression levels of two E2F targets, Cyclin A and Cyclin E. RNAseq data were obtained from TCGA data for 111 laryngeal tumors, and we compared RNA levels of Cyclin A and Cyclin E for 75 tumors with CDKN2A hypomethylation and 36 tumors with CDKN2A hypermethylation (Fig. S5). We found no significant differences in Cyclin A or Cyclin E RNA expression between the two groups. While these analyses did not directly measure the functionality of INK4a, the results suggest normal E2F activity for both groups of laryngeal tumors.

### RNA expression of INK4b is also associated with CDKN2A nonpromoter methylation

There is a possibility that the association between CDKN2A methylation and RNA expression may be a consequence of chromatin conformation with more of a regional effect on gene expression. As a preliminary test for this hypothesis, we examined the RNA expression for an adjacent locus (CDKN2B) found at chromosome 9p21. We assessed RNA levels for the INK4b gene, which is transcribed from the CDKN2B locus, in both MMC and TCGA cohorts. For the MMC samples, RNA levels for INK4b were obtained from the Illumina expression array, and found to be significantly increased in tumors with CDKN2A hypermethylation (Wilcoxon rank‐sum, *P* = 0.015; Fig. S6A). For TCGA samples, RNAseq data were collected for INK4b, which also showed a significant increase in INK4b RNA expression for tumors with CDKN2A hypermethylation (Wilcoxon rank‐sum, *P* < 0.0001; Fig. S6B). Our finding of increased INK4b RNA levels in tumors with nonpromoter CDKN2A hypermethylation suggests an open chromatin conformation for at least a portion of the 9p21 locus.

### CDKN2A methylation/expression is associated with locoregional control

In order to examine the relationship between CDKN2A methylation/expression and clinical outcome, we worked with collaborators at the University of Pittsburgh [BD and JRG], Vanderbilt University [JL], and the University of North Carolina [NH], to retrieve clinical outcome data for a subset of 40 LXSCC patients included in the TCGA (Table S4)**.** Using the same expression cutoffs established for the initial TCGA analyses, we identified 24 hypomethylated and 16 hypermethylated tumors (Fig. S7). While the TCGA subcohort was more likely to be male, Caucasian and non‐Hispanic, and less likely to be HPV positive compared to our MMC cohort, no significant associations were seen between nonpromoter CDKN2A methylation and clinicopathologic factors. RNA levels of ARF and INK4a were significantly higher in the hypermethylated vs. hypomethylated tumors from this subgroup (Wilcoxon rank‐sum test *P* < 0.0001 and *P* = 0.005, respectively).

**Figure 5:**
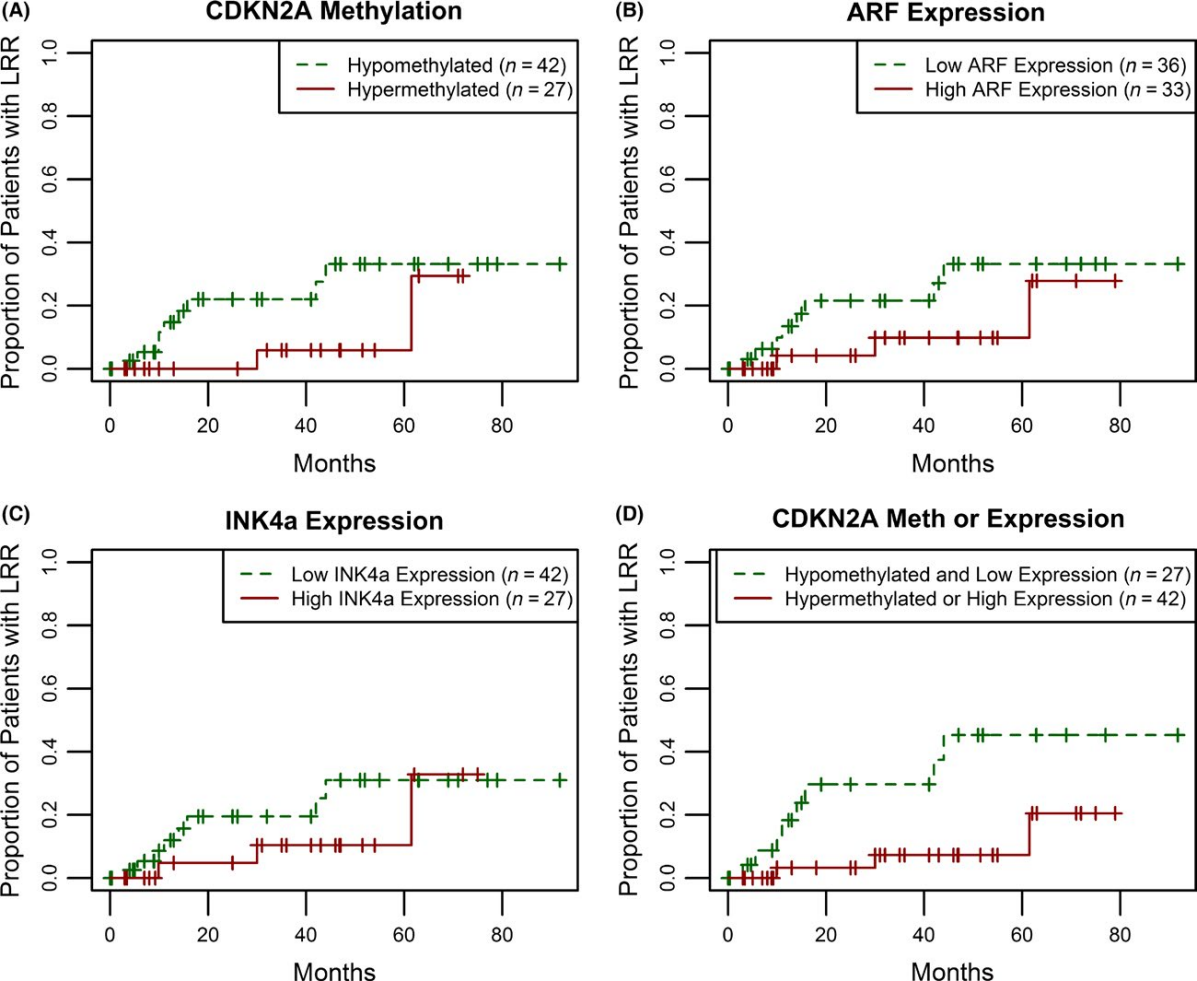
Increased risk of local regional recurrence is associated with CDKN2A hypomethylation and low RNA expression. Recurrence was assessed for 69 LXSCC patients in the combined MMC, Pittsburgh, Vanderbilt and UNC cohort. (A) A Kaplan–Meier was generated for 42 hypomethylated (green, dashed line) and 27 hypermethylated tumors (red, solid line). The plotted results indicate a decreased risk of recurrence in the hypermethylated group (Log‐rank, *P* = 0.11). (B) Patients were divided by ARF expression. The Kaplan–Meier was generated comparing 36 tumors with low ARF expression (green, dashed line) and 33 tumors with high ARF expression (red, solid line). High ARF expression was defined as a relative expression level ≥49 (∆∆CT) for the MMC cohort and ≥171 (RPKM) for the two TCGA cohorts. The results indicate a decreased risk of recurrence in the tumors with high ARF expression (Log‐rank test, *P* = 0.18). (C) Patients were divided by levels of INK4a expression. The Kaplan–Meier was generated to compare 42 tumors with low INK4a expression (green, dashed line) and 27 tumors with high INK4a expression (red, solid line). Here high INK4a expression was defined as a relative expression level ≥166 (∆∆CT) for the MMC cohort and ≥35 (RPKM) for the two TCGA cohorts. High INK4a RNA expression alone did not contribute to a decreased risk for LRR (Log‐rank, *P* = 0.31). (D) Patients were divided by CDKN2A methylation, but tumors with either high ARF or high INK4a were moved to the hypermethylated group. The Kaplan–Meier was generated comparing 27 hypomethylated tumors with low levels of CDKN2A expression (green, dashed line) and 42 tumors with either hypermethylation or high levels of CDKN2A expression (red, solid line). The results indicate a significant decreased risk of LRR in tumors with CDKN2A hypermethylation, high ARF expression, or high INK4a expression (Log‐rank, *P* = 0.01). MMC, montefiore medical center.

To evaluate the relationship between nonpromoter CDKN2A methylation, RNA expression and tumor progression, we assessed the risk of locoregional recurrence (LRR) for the combined cohort of (*n* = 69) LXSCC patients from MMC, Pittsburgh, Vanderbilt, and UNC (median follow‐up of 26 months). Figure 5A shows the Kaplan–Meier plots for LRR comparing LXSCC patients with respect to nonpromoter CDKN2A methylation status. Patients with CDKN2A hypermethylation in the tumor had a lower risk of LRR when compared to those with hypomethylated tumors, although the results were not significant (Log‐rank test *P* = 0.11). We observed similar differences when we assessed risk of LRR by CDKN2A transcript levels using median cutoff levels for ARF and INK4a RNA, although these were also not significant (Log‐rank test *P* = 0.18 for low vs. high ARF expression; Fig. 5B, and *P* = 0.31 for INK4A expression; Fig. 5C). When we further classified patients based on their combined tumor CDKN2A locus methylation/expression status, comparing patients with either hypermethylated CDKN2A or high RNA levels of ARF/INK4a to those with CDKN2A hypomethylation and low ARF+INK4a expression, we found a stronger association with risk of LRR (Log‐rank test *P* = 0.01; Fig. 5D).

To assess if CDKN2A hypermethylation and high ARF/INK4a expression was associated with lower risk of LRR after adjustment for confounding factors, multivariable cox proportional hazard regression models were constructed. After backward selection to identify significant confounders, a model adjusting for treatment and other relevant/significant clinicopathologic factors (e.g., age, gender, and nodal status at diagnosis) showed that LXSCC patients with nonpromoter CDKN2A hypermethylation, high ARF expression or high INK4a expression were significantly less likely to develop a LRR compared to patients lacking both nonpromoter CDKN2A methylation and RNA expression (adjusted hazard ratio = 0.21, 95% CI: 0.05–0.81; Table 1). Given the potential impact of treatment modalities on locoregional control, we also assessed the subset (*n* = 48) of patients who received surgery as a component of their treatment, which was the most commonly used modality (Fig. S8). As with the expanded cohort, detection of nonpromoter CDKN2A hypermethylation or high ARF/INK4A expression was associated with a significantly lower risk of LRR (Log‐rank test, *P* = 0.01). Our statistical analyses on clinical outcome indicate that a combined marker of CDKN2A methylation or expression is associated with a decreased risk of LRR for LXSCC.

**Table 1:** Multivariable cox regression model evaluating the association between CDKN2A nonpromoter methylation or expression and time to locoregional recurrence

Variable	*N*	Hazard Ratio	95% CI	*P*‐value
Methylated or high expression				
Negative	25	–		
Positive	41	0.21	(0.05, 0.81)	0.024
Age				
<60 years old	28	–		
≥ 60 years old	38	0.55	(0.12, 2.44)	0.430
Gender				
Male	48	–		
Female	18	0.30	(0.03, 2.76)	0.290
Nodal metastases				
N0	32	–		
N+	34	1.17	(0.28, 4.89)	0.830
Treatment				
Unimodality^a^	28	–		
Surgery + adjuvant^b^	25	1.48	(0.27, 8,13)	0.650
Chemo‐radiation	13	5.00	(0.91, 27.43)	0.064

Model stratified on institution (MMC or TCGA). Hazard ratios were mutually adjusted for all other variables shown.

a Unimodality, radiation alone or surgery alone.

b Adjuvant, postoperative radiation or chemoradiation.

“–”reference category.

CI, confidence interval.

## Discussion

In this study, we demonstrate that methylation of the terminal intron/exon of the CDKN2A tumor suppressor gene in LXSCC is associated with increased expression of CDKN2A transcripts, ARF and INK4a, which are known to stabilize cell cycle proteins p53 and RB, respectively 19. In addition, we found that hypermethylation and transcription of this locus was significantly associated with improved locoregional control in LXSCC.

We previously identified a significant relationship between downstream CDKN2A hypermethylation (at the same locus) and increased RNA expression that was also correlated with HPV infection in OPSCC 6. Whereas HPV is prevalent in OPSCC, LXSCC is not typically associated with HPV infection 20. Despite this, we observed a significant proportion of LXSCC exhibited hypermethylation of the same nonpromoter region of CDKN2A, and increased levels of RNA expression, suggesting that the association was not limited to HPV‐associated disease.

The CDKN2A locus is frequently altered in head and neck cancer, either through complete deletion of both transcripts or microdeletion of exons specific to ARF and/or INK4A, or through mutation producing nonfunctional proteins 10. When we compared the genetic and epigenetic data from TCGA, we found that while CDKN2A was frequently mutated in LXSCC tumors, the mutations occurred exclusively within exon 1*α* (which is unique to INK4a) and exon 2, which is shared by both transcripts and essential for ARF‐mediated MDM2 nucleolar translocation 21. No mutations were found within exon 1*β*, which is unique to ARF. In addition, a number of tumors with high CDKN2A expression also exhibited a loss of one copy of CDKN2A. This is of relevance since ARF cannot stabilize p53 without both alleles 22, and combined with exon 2 mutations, suggests the presence of a nonfunctional ARF‐MDM2‐p53 pathway. Furthermore, when we looked at the RNA expression of two downstream targets of the INK4a‐CDK‐Rb‐E2F pathway, Cyclin A and Cyclin E 23, 24, we found similar levels in tumors independent of CDKN2A methylation status. This suggests the presence of active E2F protein despite high levels of INK4a RNA.

Whereas loss of the Rb and p53 pathways may drive tumorigenesis in LXSCC, high levels of ARF RNA may enhance patient response to treatment by inducing apoptosis in tumor cells independent of p53 25, 26. In particular, the overexpression of ARF exon 1*β* alone may be sufficient to induce p53‐indepent apoptosis 26. Since exon 1*β* was found to be intact in all of TCGA LXSCC samples, we infer that tumors with high levels of ARF RNA may be generating some level of tumor suppression despite the absence of an intact p53 pathway, which improved locoregional control.

Unlike promoter methylation, which is associated with transcriptional silencing of genes 27, CpG island hypermethylation in nonpromoter regions has been associated with transcriptional activation 28. CpG methylation patterns can affect protein‐DNA binding interactions and alter chromatin structures that control corresponding gene expression 29. By altering protein‐DNA interactions, such as methylation‐sensitive CTCF protein binding, CpG methylation can play a role in chromatin looping 30, resulting in open and active regional chromatin transcriptional regulation. CTCF protein binding within the CDKN2A/B locus (including at a site adjacent to our area of interest) has previously been associated with decreased ARF, INK4a, and INK4b transcription 31. RNA expression of INK4b was associated with CDKN2A nonpromoter methylation in both the MMC and TCGA cohorts, suggesting a region of open chromatin and active transcription.

Although we did not investigate the potential mechanisms for the associations between CDKN2A nonpromoter methylation and RNA expression in LXSCC tumors, we did validate our findings using the publicly available TCGA data and were able to establish a clinical relevance for the observations. Using TCGA data, we found that neither CDKN2A metylation status nor RNA expression was reflective of CNV except in the case of complete deletion (<1 copy), which made up less than half the cases without CDKN2A methylation and high RNA expression. Therefore, it is unlikely that the observed associations were driven by CNV. It should be noted that the combination of our institutional samples with TCGA necessitated comparing data from different methylation and expression platforms, which required statistical adjustment to compare high versus low methylation and RNA expression. However, given that the same relative associations were found across multiple assays and specimens from the different studies, this suggests our findings are robust. Furthermore, the association between CDKN2A nonpromoter methylation or expression and LRR remained significant after adjustment for clinicopathologic factors.

It should also be noted that this initial study did not assess correlation with protein levels (other than clinical p16 staining by IHC). While we did not see a significant association between p16 protein expression and prognosis in non‐OPSCC 8, others have shown p16(INK4a) expression to be associated with improved progression‐free survival in LXSCC 32. Further independent validation is mandated to establish the clinical utility of our findings, and future studies will also need to include an assessment of p14(ARF) protein expression.

In conclusion, we show that nonpromoter hypermethylation of the CDKN2A downstream locus correlates with increased ARF and INK4a mRNA expression, and that activation of these CDKN2A transcripts is associated with improved locoregional control in LXSCC. Additional work is needed to elucidate the exact mechanism driving increased nonpromoter CDKN2A methylation and expression, and how this affects response to treatment in LXSCC.

## Conflict of Interest

The authors report no conflicts of interest.

## Supporting information


**Figure S1.** ARF and all CDKN2A variants are overexpressed in laryngeal tumors with downstream CDKN2A methylation.

**Figure S2.** ARF expression is associated with CDKN2A downstream methylation. Relative ARF expression levels were plotted against M‐values for each CpG assayed in the downstream region of the CDKN2A locus.

**Figure S3.** INK4a expression is associated with nonpromtoer CDKN2A methylation.

**Figure S4**. ARF and INK4a expression are associated with CDKN2A nonpromoter methylation in TCGA laryngeal tumors.

**Figure S5.** Expression of Cyclin A and Cyclin E are similar between hypomethylated and hypermethylated laryngeal tumors in TCGA.

**Figure S6.** INK4b expression is increased in hypermethylated laryngeal tumors from MMC and TCGA Cohorts.

**Figure S7.** ARF and INK4a expression are increased in hypermethylated laryngeal tumors from TCGA.

**Figure S8.** Nonpromoter CDKN2A hypomethylation and low ARF/INK4a expression in tumors is associated with an increased risk of local regional recurrence in laryngeal cancer patients treated with surgery.

**Table S1.** Clinical data for the montefiore medical center cohort.

**Table S2.** Clinical data for the patients from the montefiore medical center cohort with qRT‐PCR data.

**Table S3.** Clinical data for TCGA laryngeal tumors.

**Table S4.** Clinical data for the University of Pittsburgh, Vanderbilt University, and University of North Carolina of Chapel Hill Cohorts.

## References

[1] Siegel, R. L. , K. D. Miller , and A. Jemal . 2016 Cancer statistics, 2016. CA Cancer J. Clin. 66:7–30. 26742998 10.3322/caac.21332

[2] Weber, R. S. , B. A. Berkey , A. Forastiere , et al. 2003 Outcome of salvage total laryngectomy following organ preservation therapy: the Radiation Therapy Oncology Group trial 91‐11. Arch. Otolaryngol. Head Neck Surg. 129:44–49. 12525193 10.1001/archotol.129.1.44

[3] Cooper, J. S. , T. F. Pajak , A. A. Forastiere , et al. 2004 Postoperative concurrent radiotherapy and chemotherapy for high‐risk squamous‐cell carcinoma of the head and neck. N. Engl. J. Med. 350:1937–1944. 15128893 10.1056/NEJMoa032646

[4] Ang, K. K. , J. Harris , R. Wheeler , et al. 2010 Human papillomavirus and survival of patients with oropharyngeal cancer. N. Engl. J. Med. 363:24–35. 20530316 10.1056/NEJMoa0912217 PMC2943767

[5] Castellsague, X. , L. Alemany , and M. Quer , et al. 2016 HPV Involvement in Head and Neck Cancers: Comprehensive Assessment of Biomarkers in 3680 Patients. J. Natl Cancer Inst. 108:djv403. 26823521 10.1093/jnci/djv403

[6] Schlecht, N. F. , M. Ben‐Dayan , N. Anayannis , et al. 2015 Epigenetic changes in the CDKN2A locus are associated with differential expression of P16INK4A and P14ARF in HPV‐positive oropharyngeal squamous cell carcinoma. Cancer Med 4:342–353. 25619363 10.1002/cam4.374 PMC4380960

[7] Sherr, C. J. 2001 The INK4a/ARF network in tumour suppression. Nat. Rev. Mol. Cell Biol. 2:731–737. 11584300 10.1038/35096061

[8] Salazar, C. R. , N. Anayannis , R. V. Smith , et al. 2014 Combined P16 and human papillomavirus testing predicts head and neck cancer survival. Int. J. Cancer 135:2404–2412. 24706381 10.1002/ijc.28876 PMC4159440

[9] Yarbrough, W. G. 2002 The ARF‐p16 gene locus in carcinogenesis and therapy of head and neck squamous cell carcinoma. Laryngoscope 112:2114–2128. 12461329 10.1097/00005537-200212000-00002

[10] Cancer Genome Atlas . 2015 N. Comprehensive genomic characterization of head and neck squamous cell carcinomas. Nature 517:576–582. 25631445 10.1038/nature14129 PMC4311405

[11] Lleras, R. A. , R. V. Smith , L. R. Adrien , et al. 2013 Unique DNA methylation loci distinguish anatomic site and HPV status in head and neck squamous cell carcinoma. Clin. Cancer Res. 19:5444–5455. 23894057 10.1158/1078-0432.CCR-12-3280 PMC3892374

[12] Mancuso, F. M. , M. Montfort , A. Carreras , A. Alibes , and G. Roma . 2011 HumMeth27QCReport: an R package for quality control and primary analysis of Illumina Infinium methylation data. BMC Res.Notes 4:546. 22182516 10.1186/1756-0500-4-546 PMC3285701

[13] Fhag, J. 2014 supraHex: an R/Bioconductor package for tabular omics data analysis using a supra‐hexagonal map. Biochem. Biophys. Res. Commun. 443:285–289. 24309102 10.1016/j.bbrc.2013.11.103 PMC3905187

[14] Wickham, H. 2009 ggplot2: Elegant Graphics for Data Analysis. Springer‐Verlag, New York.

[15] Sobin L., M. G. , Wittekind C., eds. 2009 TNM classification of malignant tumours. 7th ed John Wiley & Sons, Inc, Hoboken, NJ.

[16] S. B. Edge, C. C. Compton , A. G. Fritz , F. L. Greene , and A. Trotti , eds. 2009 American Joint Committee on Cancer Staging Manual. 7th ed Springer, New York.

[17] Greenland, S. , and J. M. Robins . 1986 Identifiability, exchangeability, and epidemiological confounding. Int. J. Epidemiol. 15:413–419. 3771081 10.1093/ije/15.3.413

[18] Grambsch TMTaPM . 2000 Modeling Survival Data: Extending the Cox Model. Springer, New York.

[19] Chin, L. , J. Pomerantz , and R. A. DePinho . 1998 The INK4a/ARF tumor suppressor: one gene–two products–two pathways. Trends Biochem. Sci. 23:291–296. 9757829 10.1016/s0968-0004(98)01236-5

[20] Torrente, M. C. , J. P. Rodrigo , M. Jr Haigentz , et al. 2011 Human papillomavirus infections in laryngeal cancer. Head Neck 33:581–586. 20848441 10.1002/hed.21421

[21] Zhang, Y. , and Y. Xiong . 1999 Mutations in human ARF exon 2 disrupt its nucleolar localization and impair its ability to block nuclear export of MDM2 and p53. Mol. Cell 3:579–591. 10360174 10.1016/s1097-2765(00)80351-2

[22] Eischen, C. M. , J. R. Alt , and P. Wang . 2004 Loss of one allele of ARF rescues Mdm2 haploinsufficiency effects on apoptosis and lymphoma development. Oncogene 23:8931–8940. 15467748 10.1038/sj.onc.1208052

[23] Girard, F. , U. Strausfeld , A. Fernandez , and N. J. Lamb . 1991 Cyclin A is required for the onset of DNA replication in mammalian fibroblasts. Cell 67:1169–1179. 1836977 10.1016/0092-8674(91)90293-8

[24] Jackson, P. K. , S. Chevalier , M. Philippe , and M. W. Kirschner . 1995 Early events in DNA replication require cyclin E and are blocked by p21CIP1. J. Cell Biol. 130:755–769. 7642695 10.1083/jcb.130.4.755 PMC2199964

[25] Hemmati, P. G. , B. Gillissen , C. von Haefen , et al. 2002 Adenovirus‐mediated overexpression of p14(ARF) induces p53 and Bax‐independent apoptosis. Oncogene 21:3149–3161. 12082630 10.1038/sj.onc.1205458

[26] Saadatmandi, N. , T. Tyler , Y. Huang , et al. 2002 Growth suppression by a p14(ARF) exon 1beta adenovirus in human tumor cell lines of varying p53 and Rb status. Cancer Gene Ther. 9:830–839. 12224024 10.1038/sj.cgt.7700505

[27] Esteller, M. . 2007 Epigenetic gene silencing in cancer: the DNA hypermethylome. Hum. Mol. Genet. 16 Spec No 1: R50–R59. 17613547 10.1093/hmg/ddm018

[28] Falzone, L. , R. Salemi , S. Travali , et al. 2016 MMP‐9 overexpression is associated with intragenic hypermethylation of MMP9 gene in melanoma. Aging (Albany NY). 8:933–944. 27115178 10.18632/aging.100951 PMC4931845

[29] Ballestar, E. , and A. P. Wolffe . 2001 Methyl‐CpG‐binding proteins. Targeting specific gene repression. Eur. J. Biochem. 268:1–6. 11121095 10.1046/j.1432-1327.2001.01869.x

[30] Kang, J. Y. , S. H. Song , J. Yun , et al. 2015 Disruption of CTCF/cohesin‐mediated high‐order chromatin structures by DNA methylation downregulates PTGS2 expression. Oncogene 34:5677–5684. 25703332 10.1038/onc.2015.17

[31] Hirosue, A. , K. Ishihara , K. Tokunaga , et al. 2012 Quantitative assessment of higher‐order chromatin structure of the INK4/ARF locus in human senescent cells. Aging Cell 11:553–556. 22340434 10.1111/j.1474-9726.2012.00809.x

[32] Chung, C. H. , Q. Zhang , C. S. Kong , et al. 2014 p16 protein expression and human papillomavirus status as prognostic biomarkers of nonoropharyngeal head and neck squamous cell carcinoma. J. Clin. Oncol. 32:3930–3938. 25267748 10.1200/JCO.2013.54.5228 PMC4251957

