# Supplementary figures and images for: Nonpromoter methylation of the CDKN2A gene with active transcription is associated with improved locoregional control in laryngeal squamous cell carcinoma
Source: Cancer Med. 2017 Jan 19;6(2):397–407. doi: 10.1002/cam4.961 (PMC5313649; doi:10.1002/cam4.961)

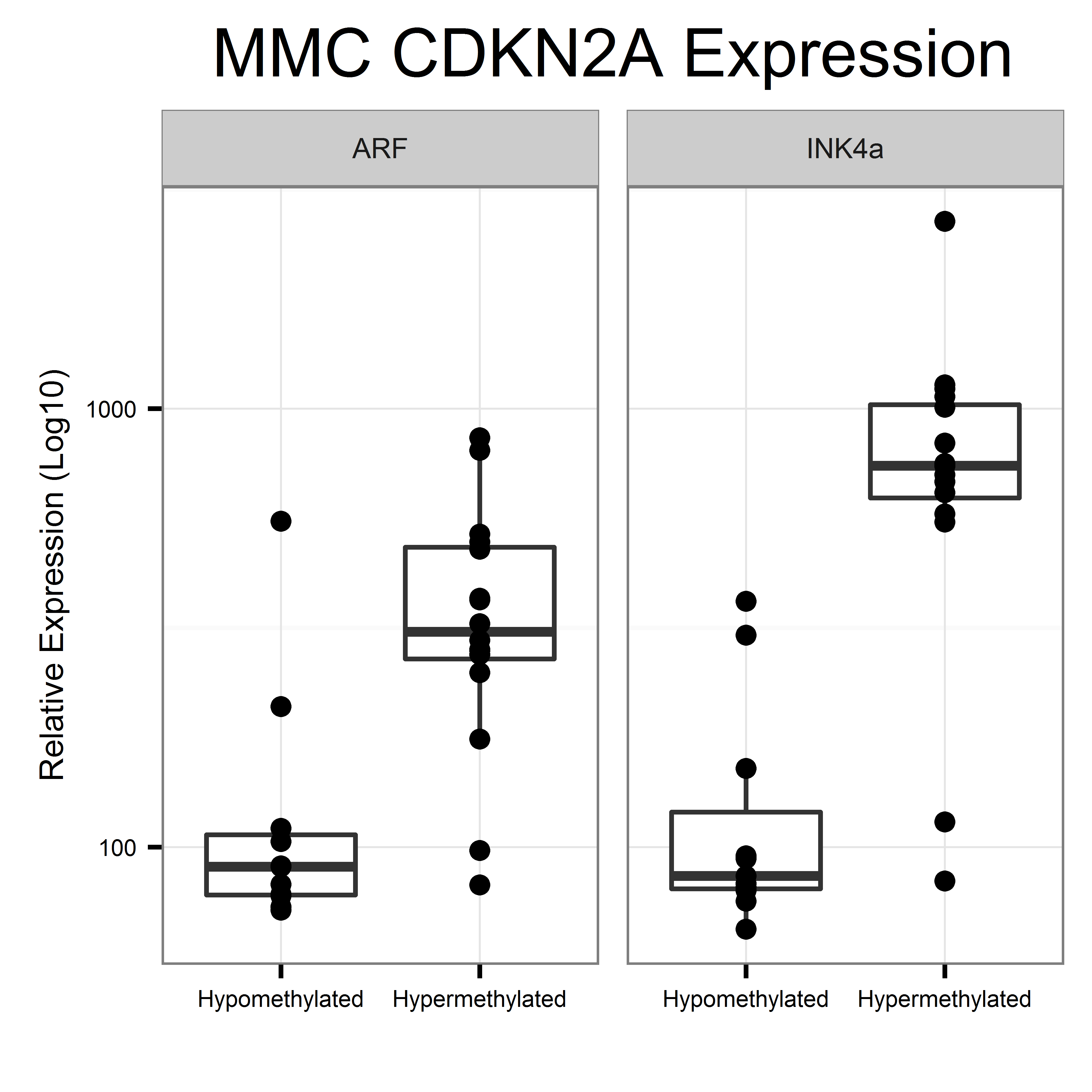

Supplement: Supplementary file 1 — Figure S1. ARF and all CDKN2A variants are overexpressed in laryngeal tumors with downstream CDKN2A methylation. [file CAM4-6-397-s001.tif]

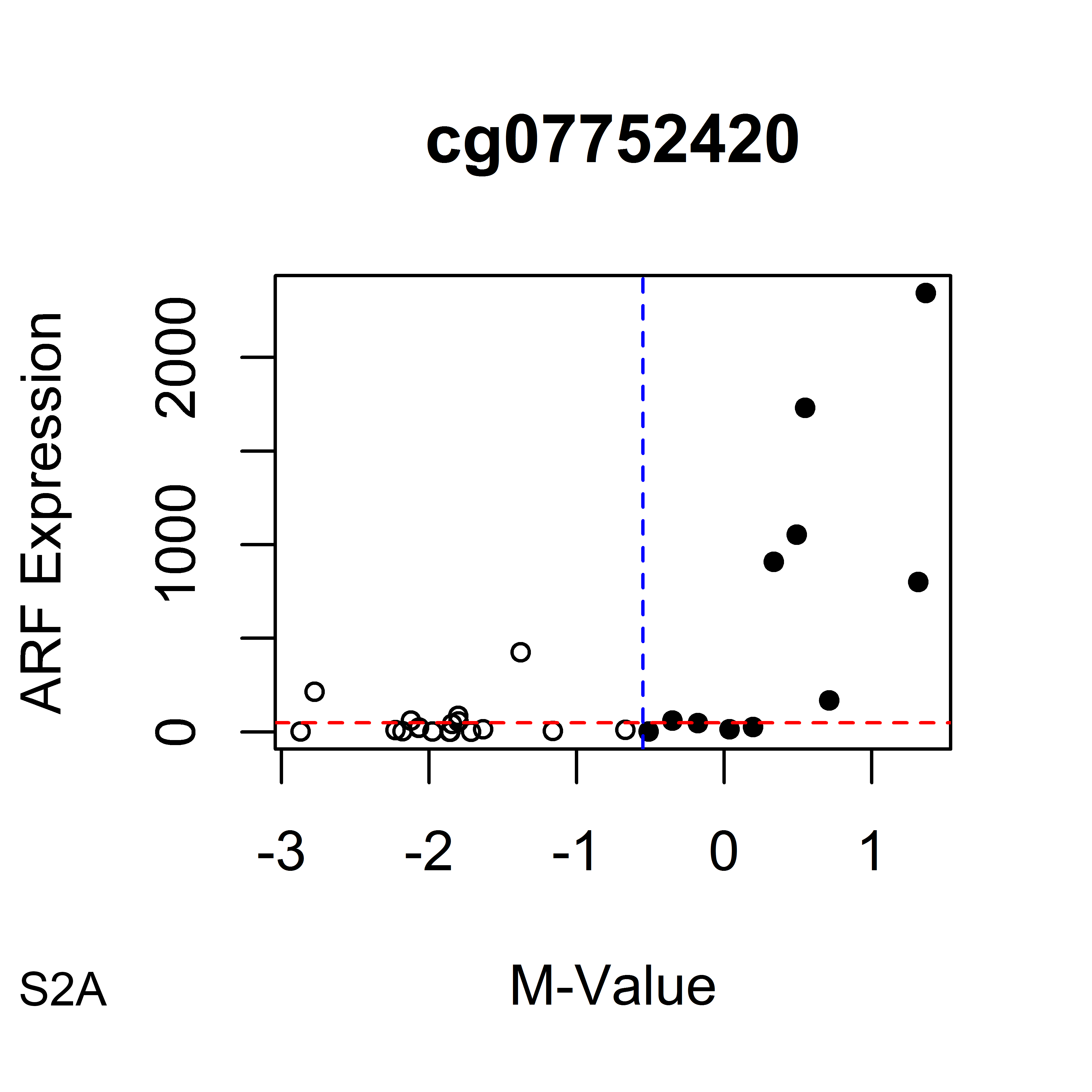

Supplement: Supplementary file 2 — Figure S2. ARF expression is associated with CDKN2A downstream methylation. Relative ARF expression levels were plotted against M‐values for each CpG assayed in the downstream region of the CDKN2A locus. [file CAM4-6-397-s002.tif]

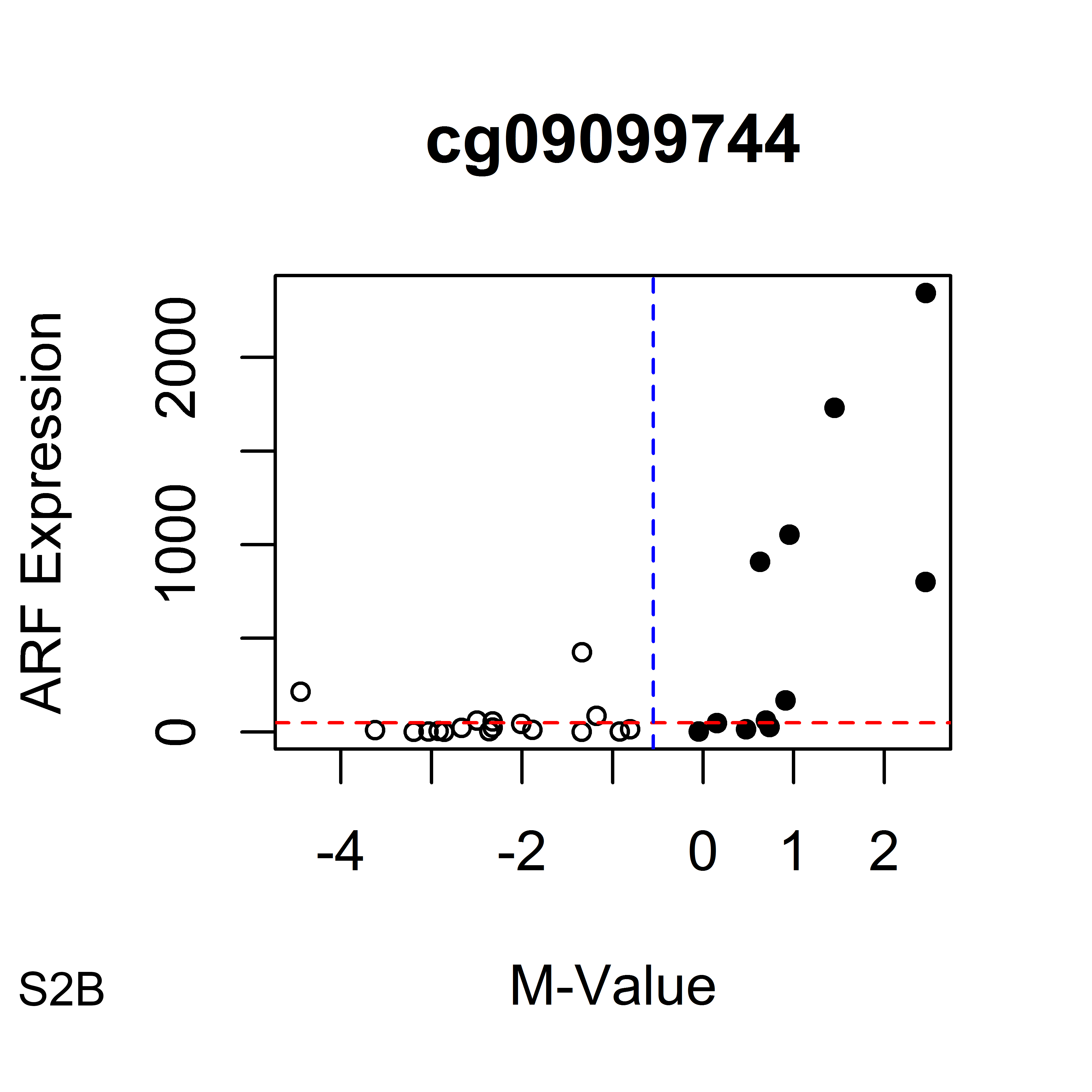

Supplement: Supplementary file 3 — Figure S3. INK4a expression is associated with nonpromtoer CDKN2A methylation. [file CAM4-6-397-s003.tif]

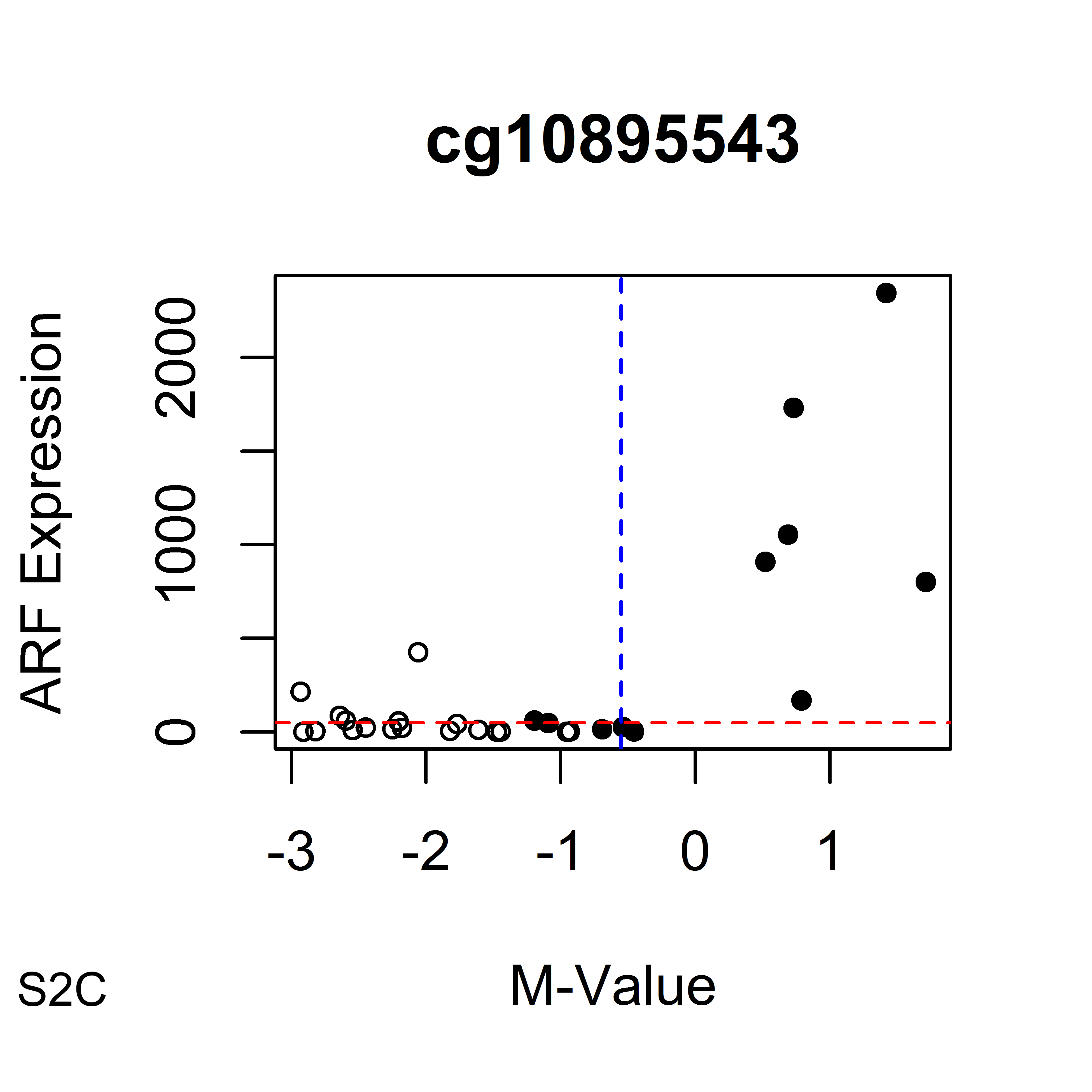

Supplement: Supplementary file 4 — Figure S4. ARF and INK4a expression are associated with CDKN2A nonpromoter methylation in TCGA laryngeal tumors. [file CAM4-6-397-s004.tif]

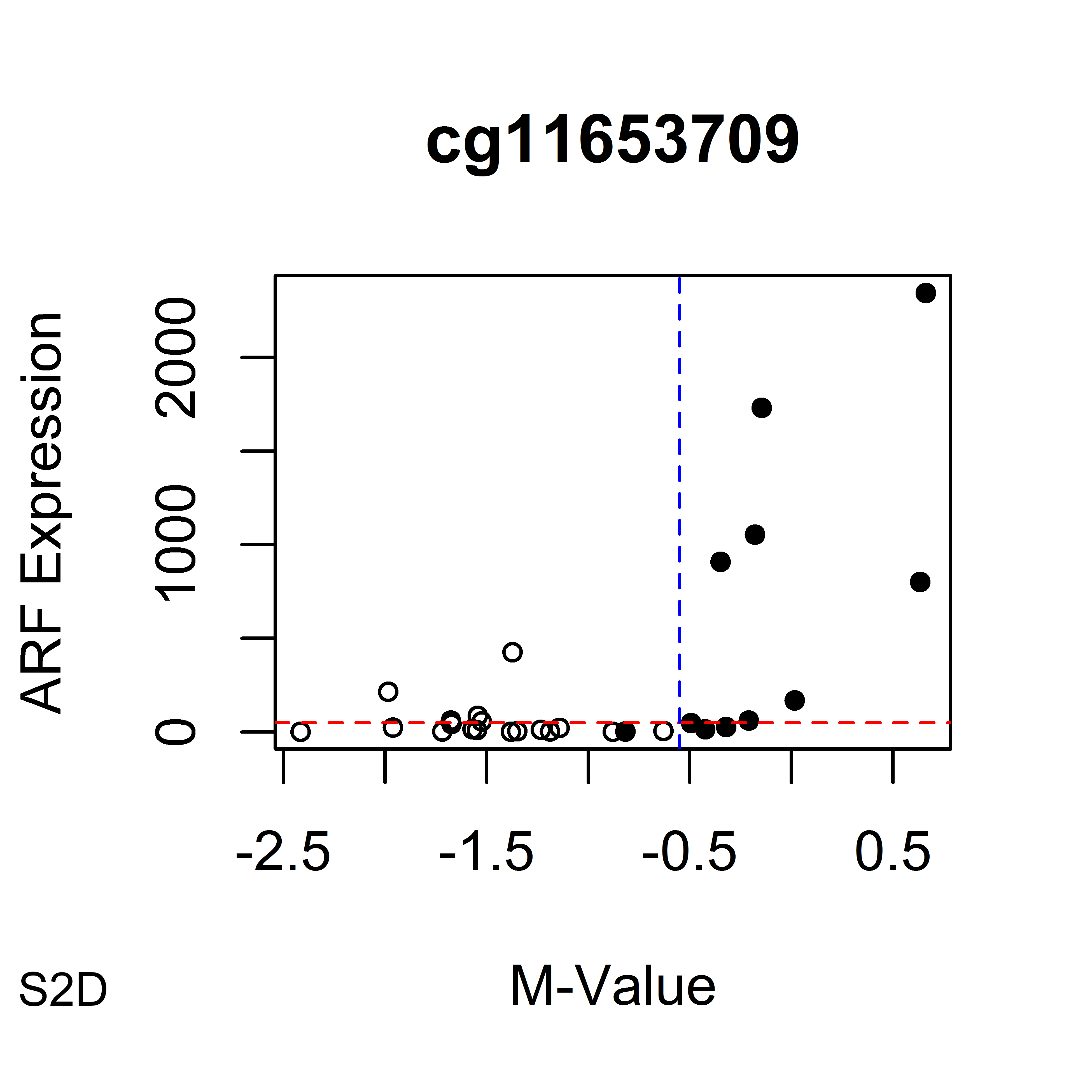

Supplement: Supplementary file 5 — Figure S5. Expression of Cyclin A and Cyclin E are similar between hypomethylated and hypermethylated laryngeal tumors in TCGA. [file CAM4-6-397-s005.tif]

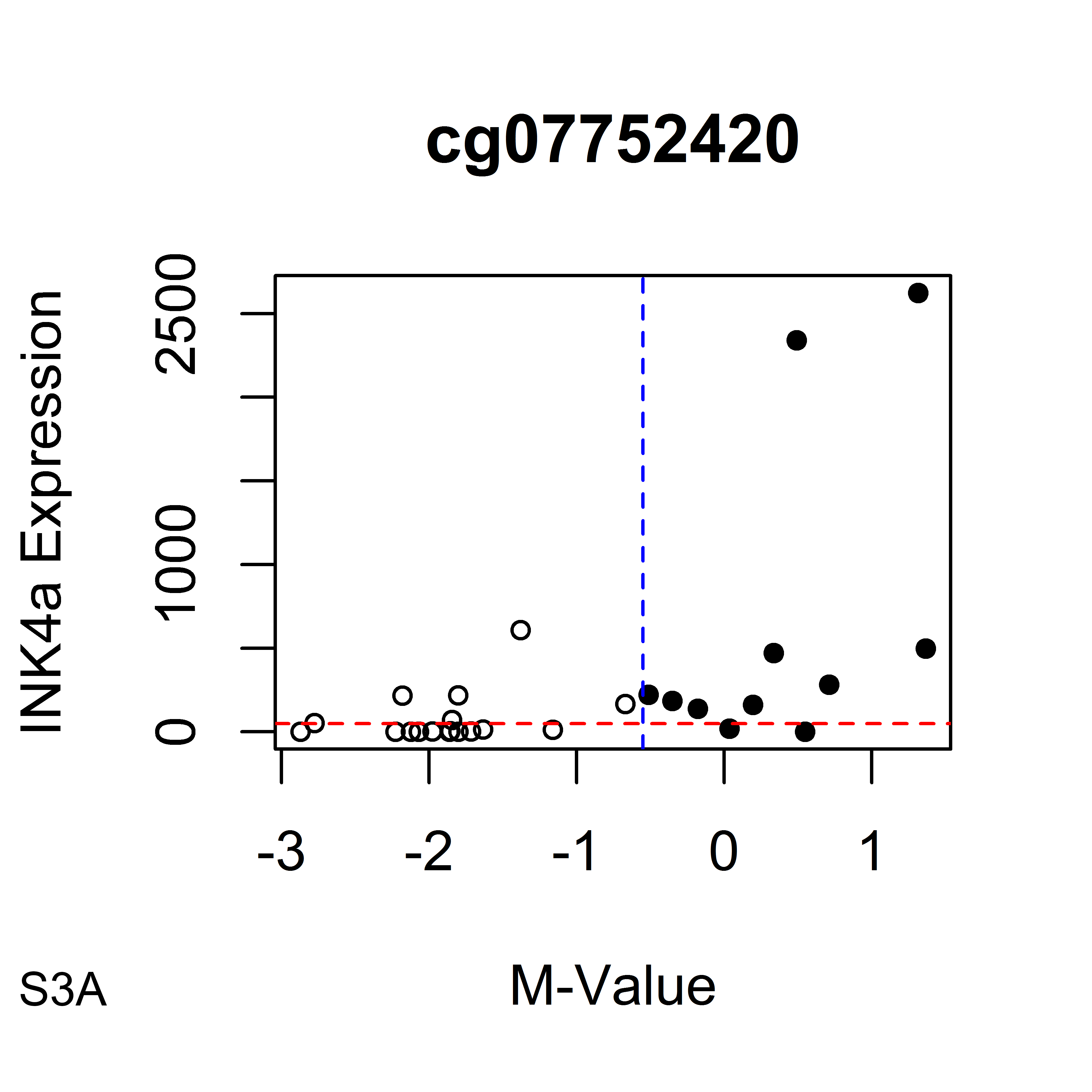

Supplement: Supplementary file 6 — Figure S6. INK4b expression is increased in hypermethylated laryngeal tumors from MMC and TCGA Cohorts. [file CAM4-6-397-s006.tif]

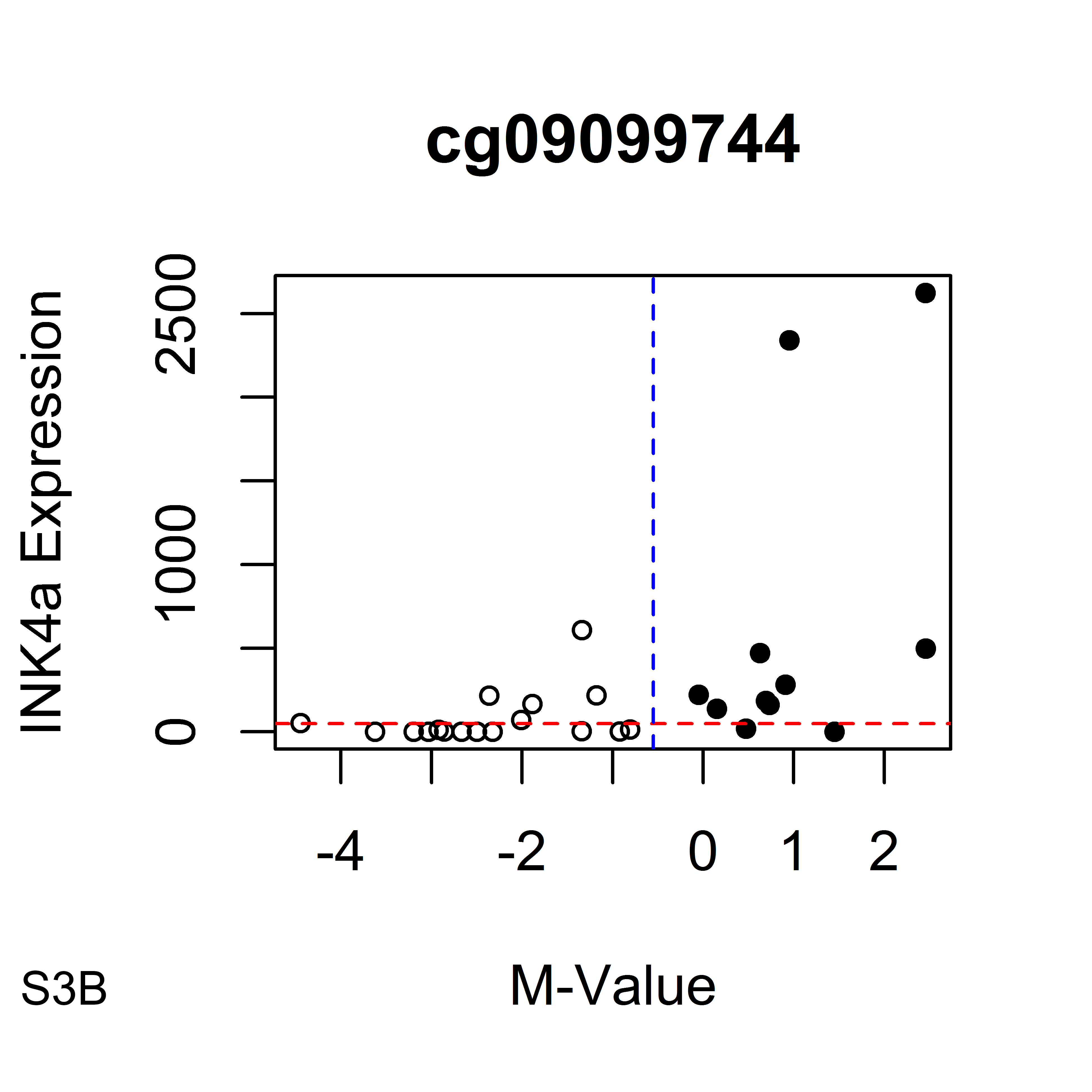

Supplement: Supplementary file 7 — Figure S7. ARF and INK4a expression are increased in hypermethylated laryngeal tumors from TCGA. [file CAM4-6-397-s007.tif]

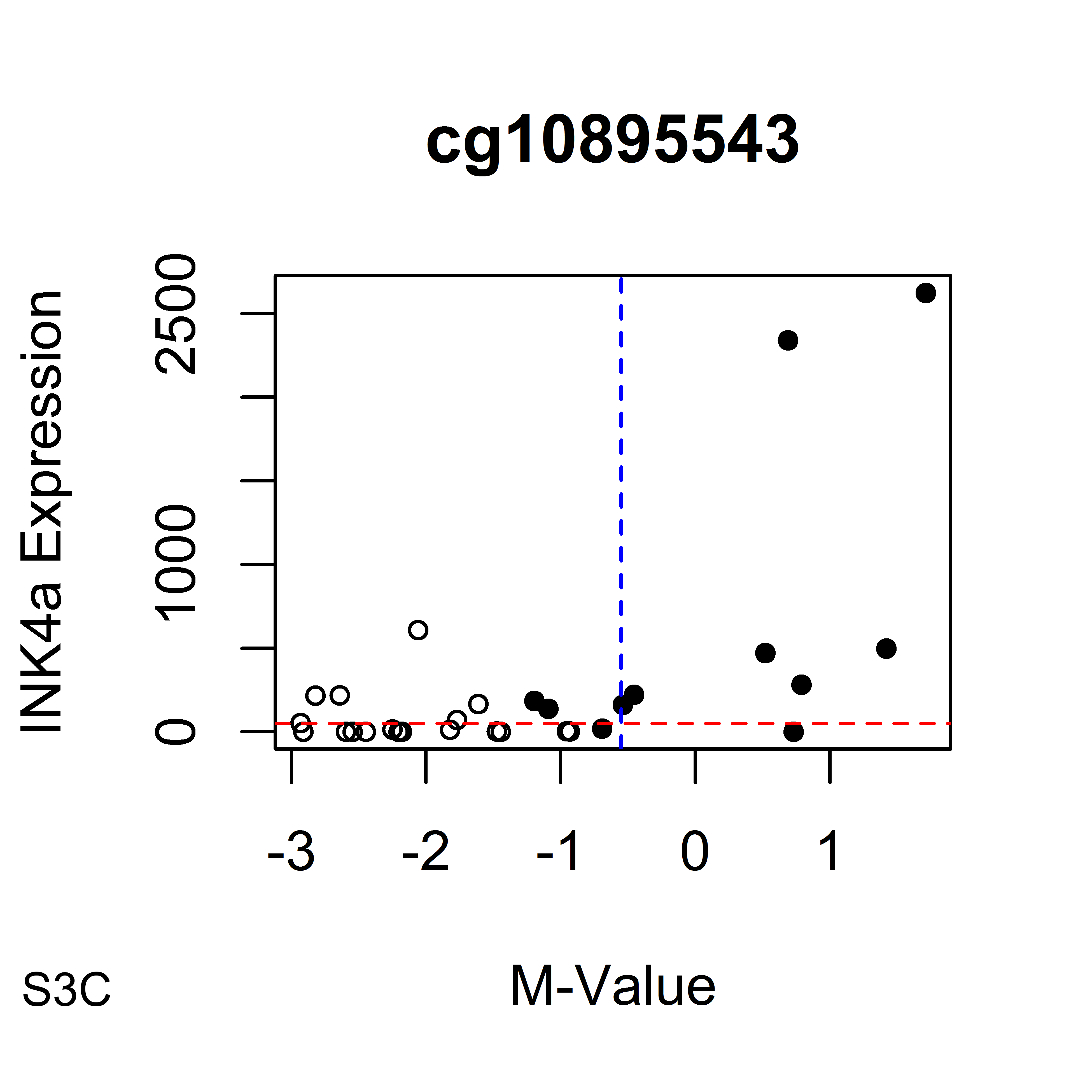

Supplement: Supplementary file 8 — Figure S8. Nonpromoter CDKN2A hypomethylation and low ARF/INK4a expression in tumors is associated with an increased risk of local regional recurrence in laryngeal cancer patients treated with surgery. [file CAM4-6-397-s008.tif]

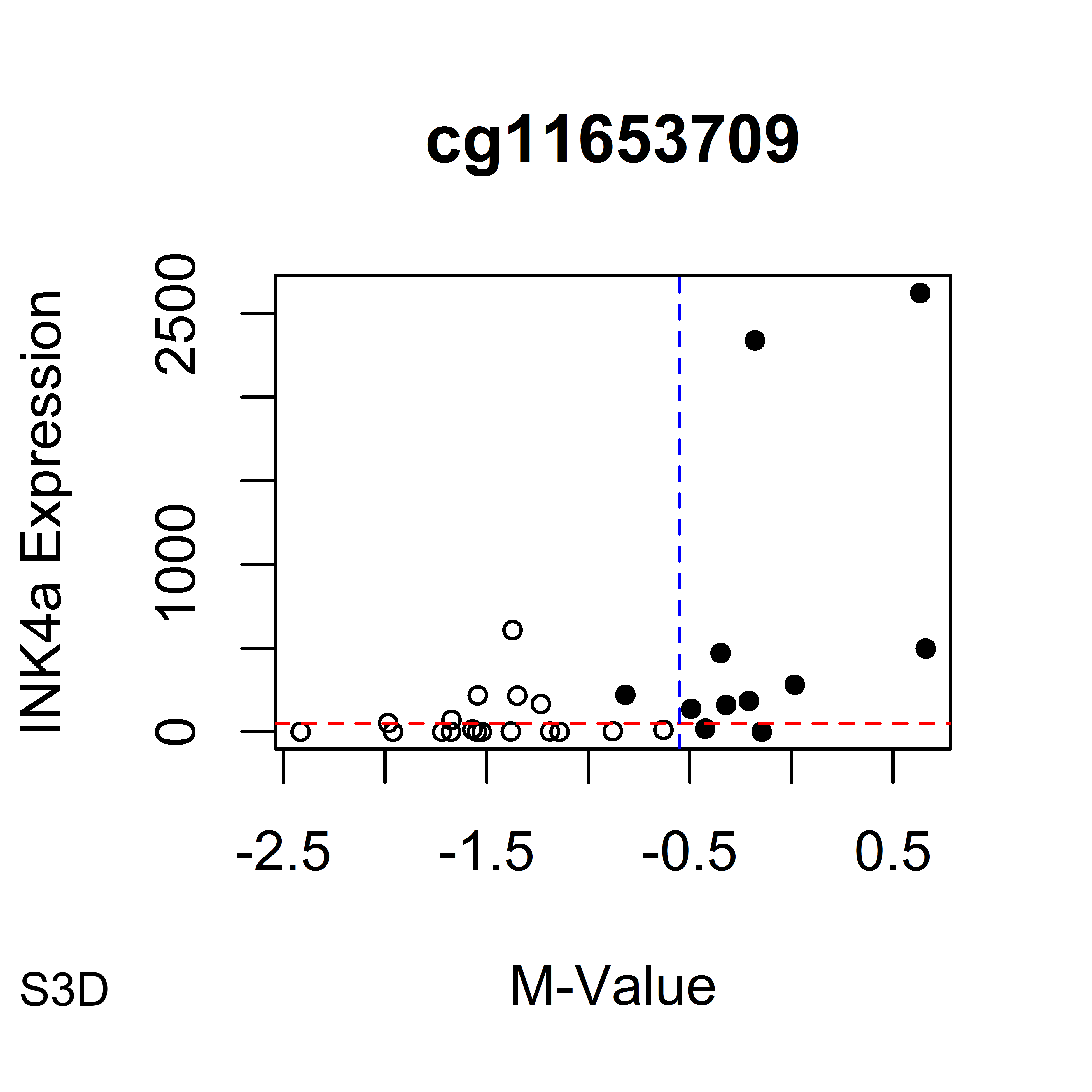

Supplement: Supplementary file 9 — Table S1. Clinical data for the montefiore medical center cohort. [file CAM4-6-397-s009.tif]

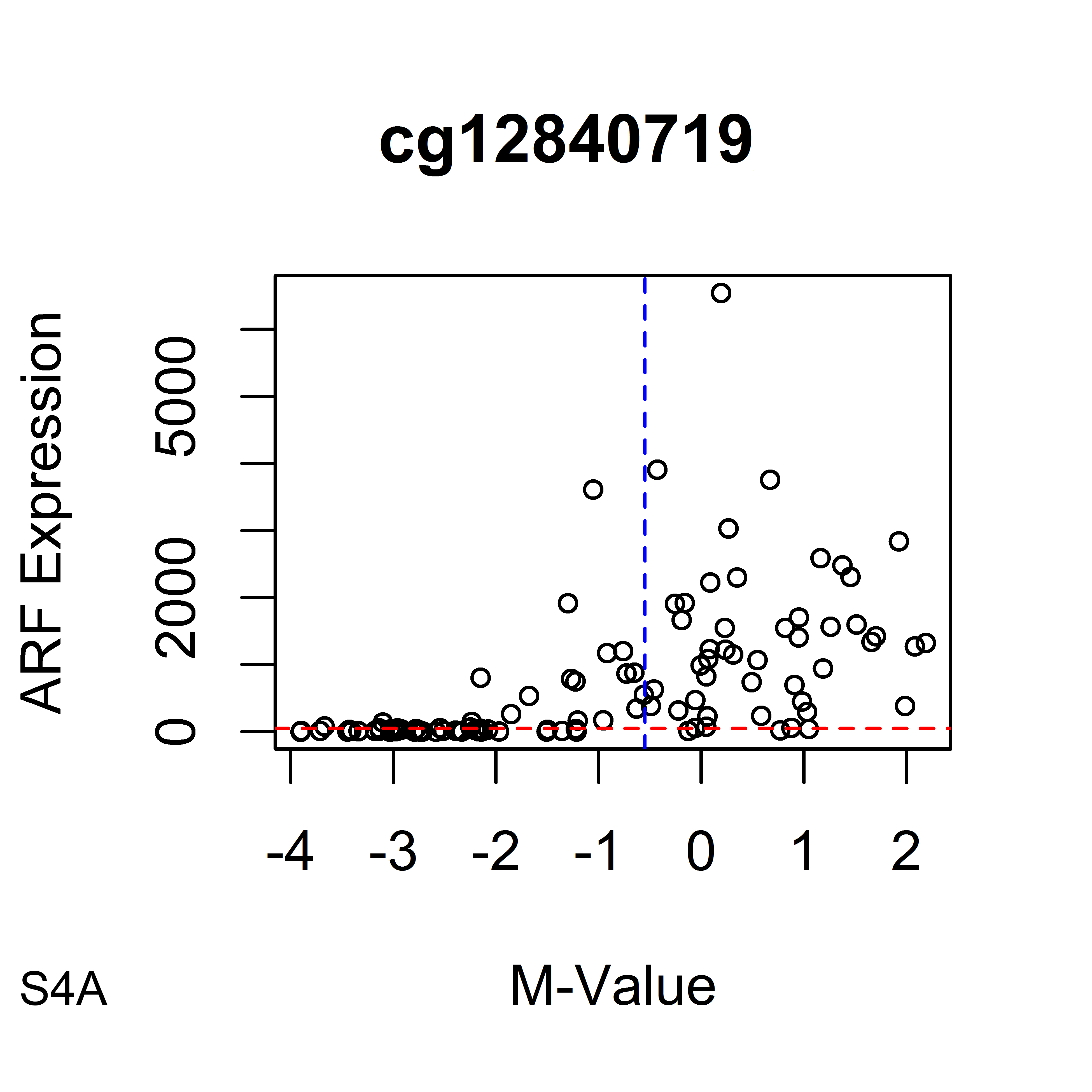

Supplement: Supplementary file 10 — Table S2. Clinical data for the patients from the montefiore medical center cohort with qRT‐PCR data. [file CAM4-6-397-s010.tif]

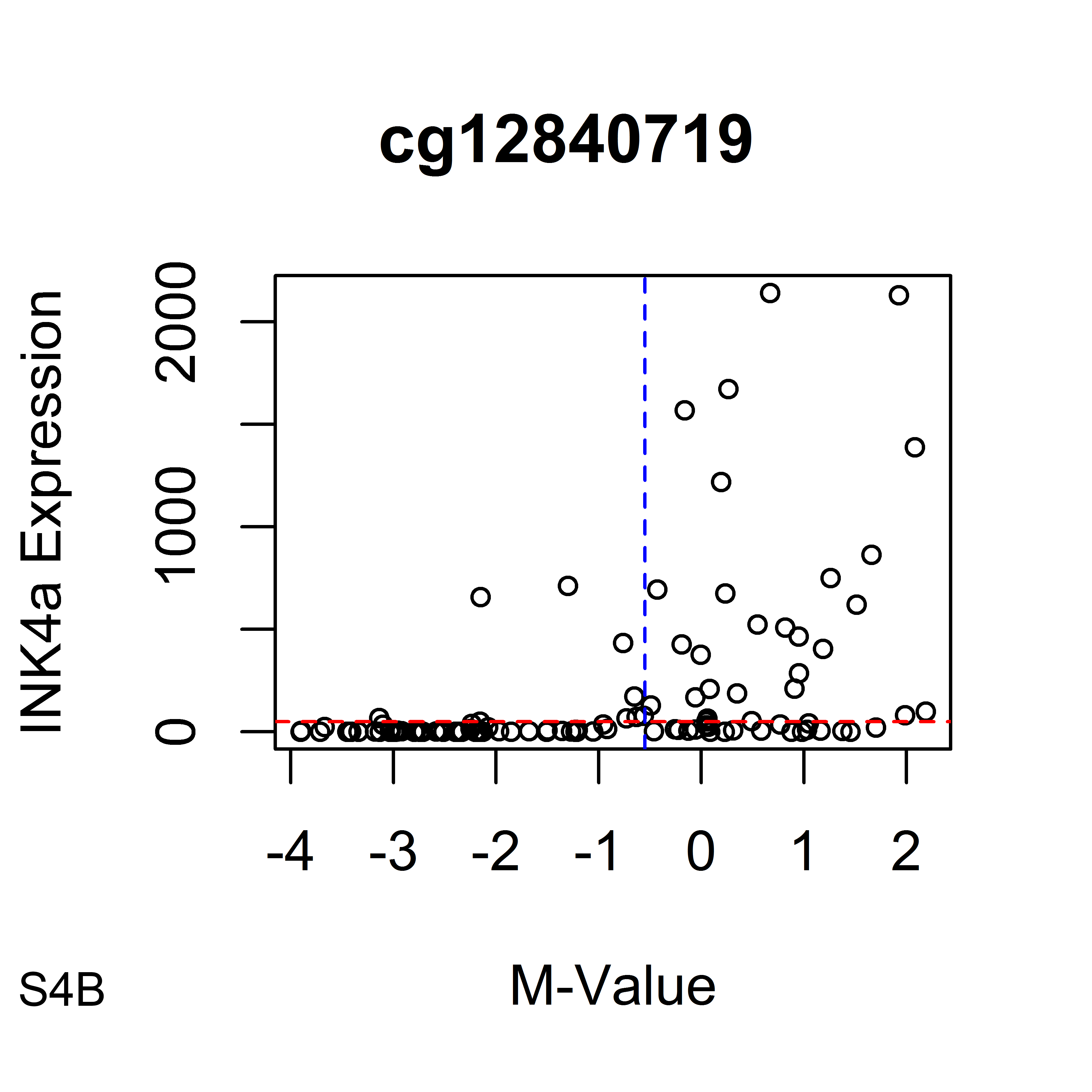

Supplement: Supplementary file 11 — Table S3. Clinical data for TCGA laryngeal tumors. [file CAM4-6-397-s011.tif]

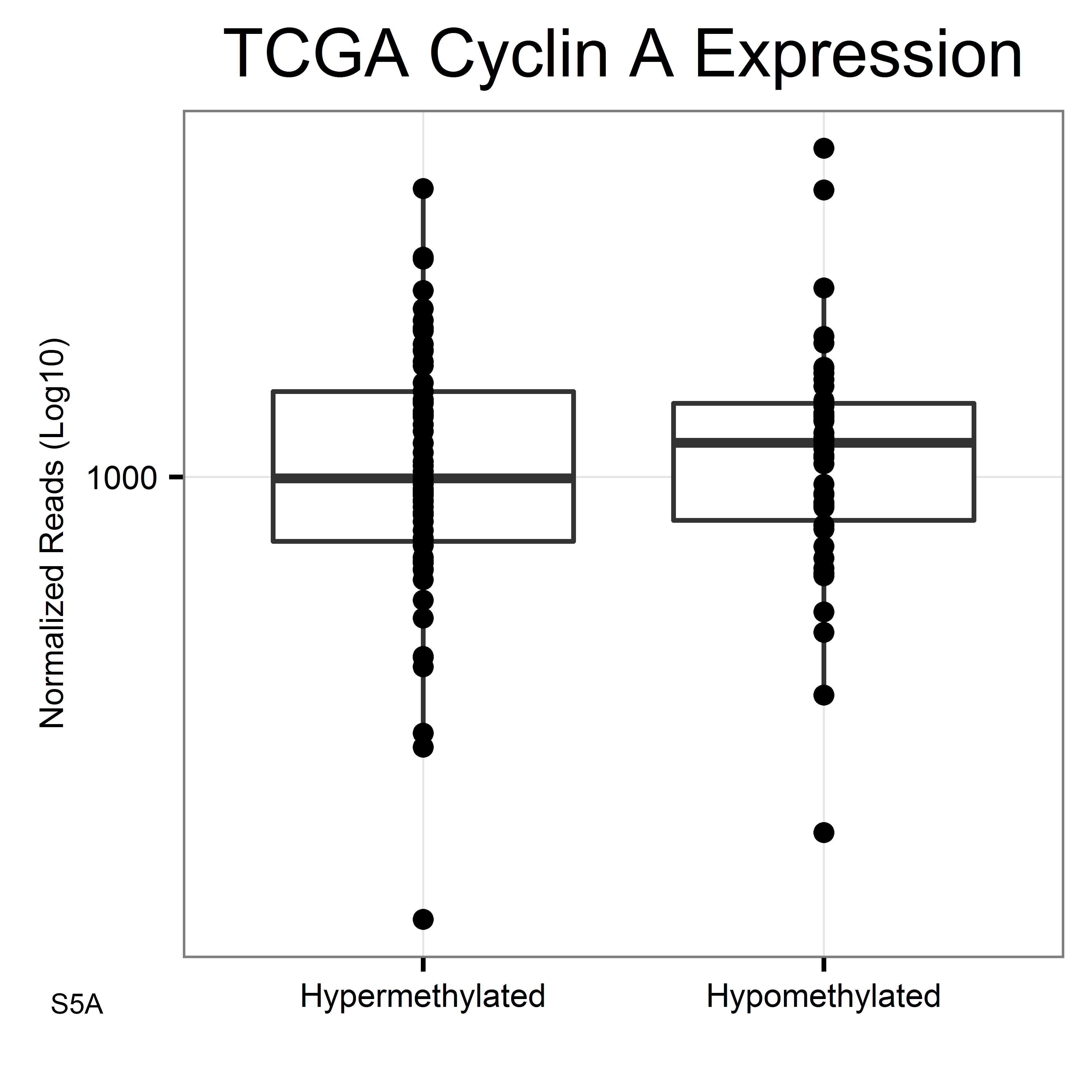

Supplement: Supplementary file 12 — Table S4. Clinical data for the University of Pittsburgh, Vanderbilt University, and University of North Carolina of Chapel Hill Cohorts. [file CAM4-6-397-s012.tif]

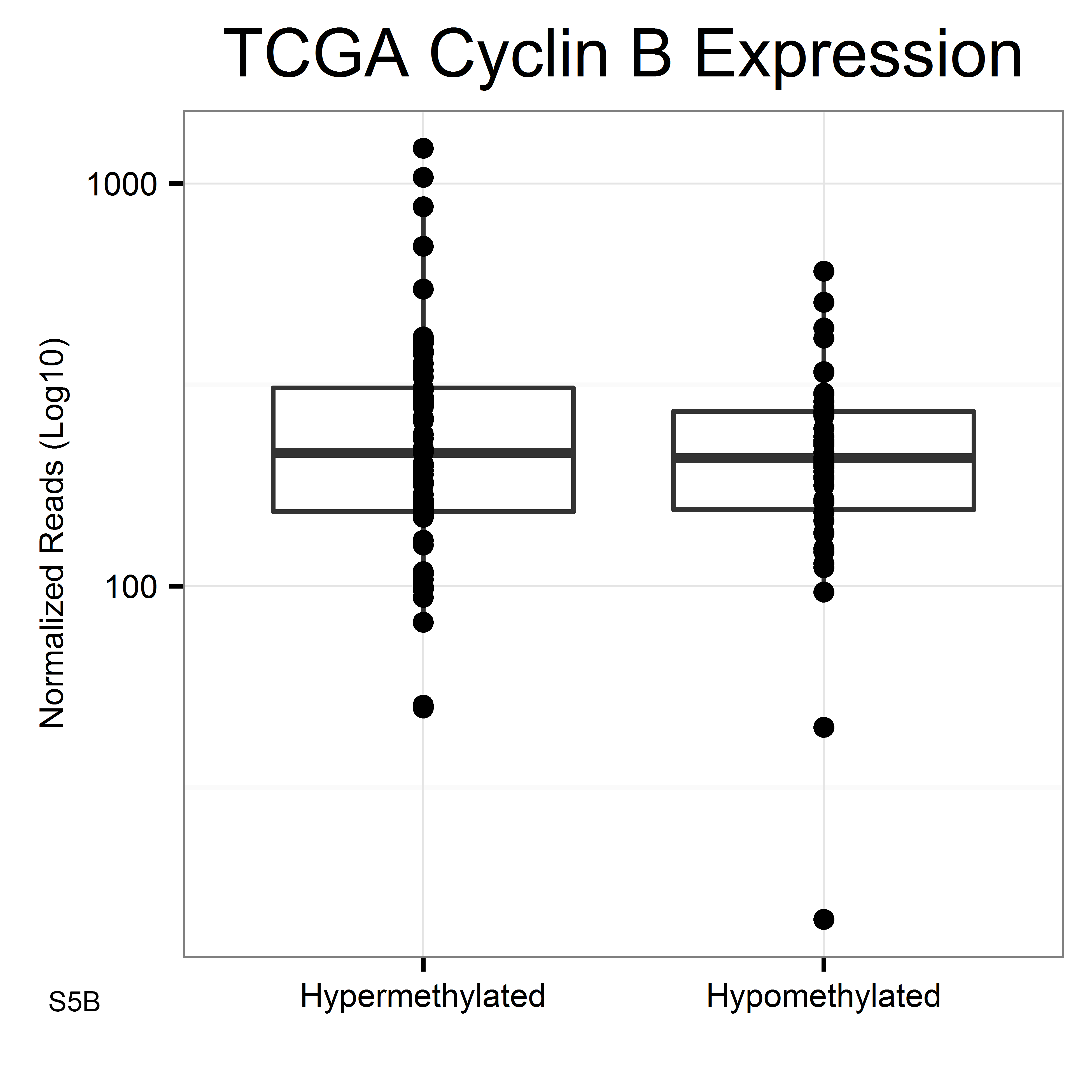

Supplement: Supplementary file 13 [file CAM4-6-397-s013.tif]

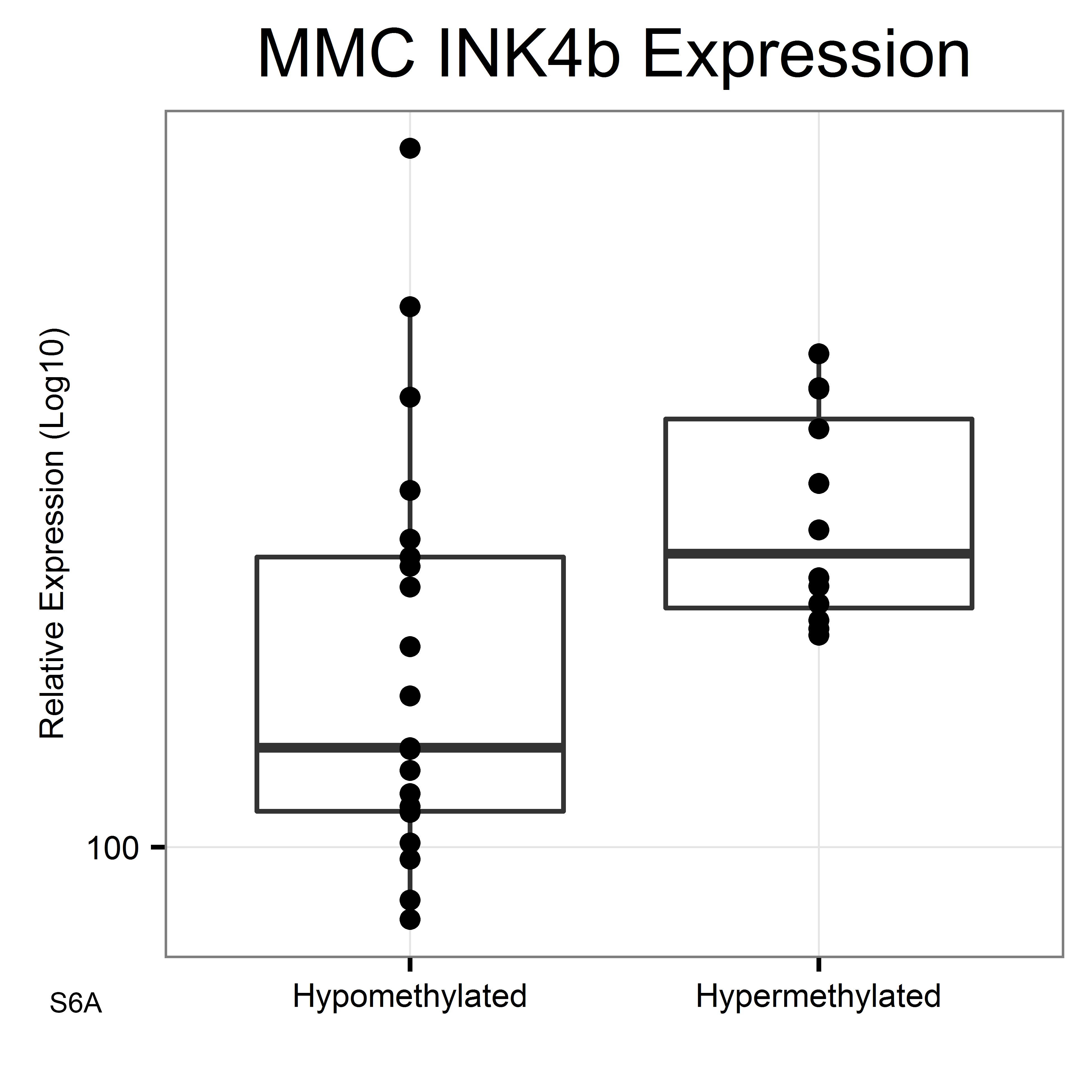

Supplement: Supplementary file 14 [file CAM4-6-397-s014.tif]

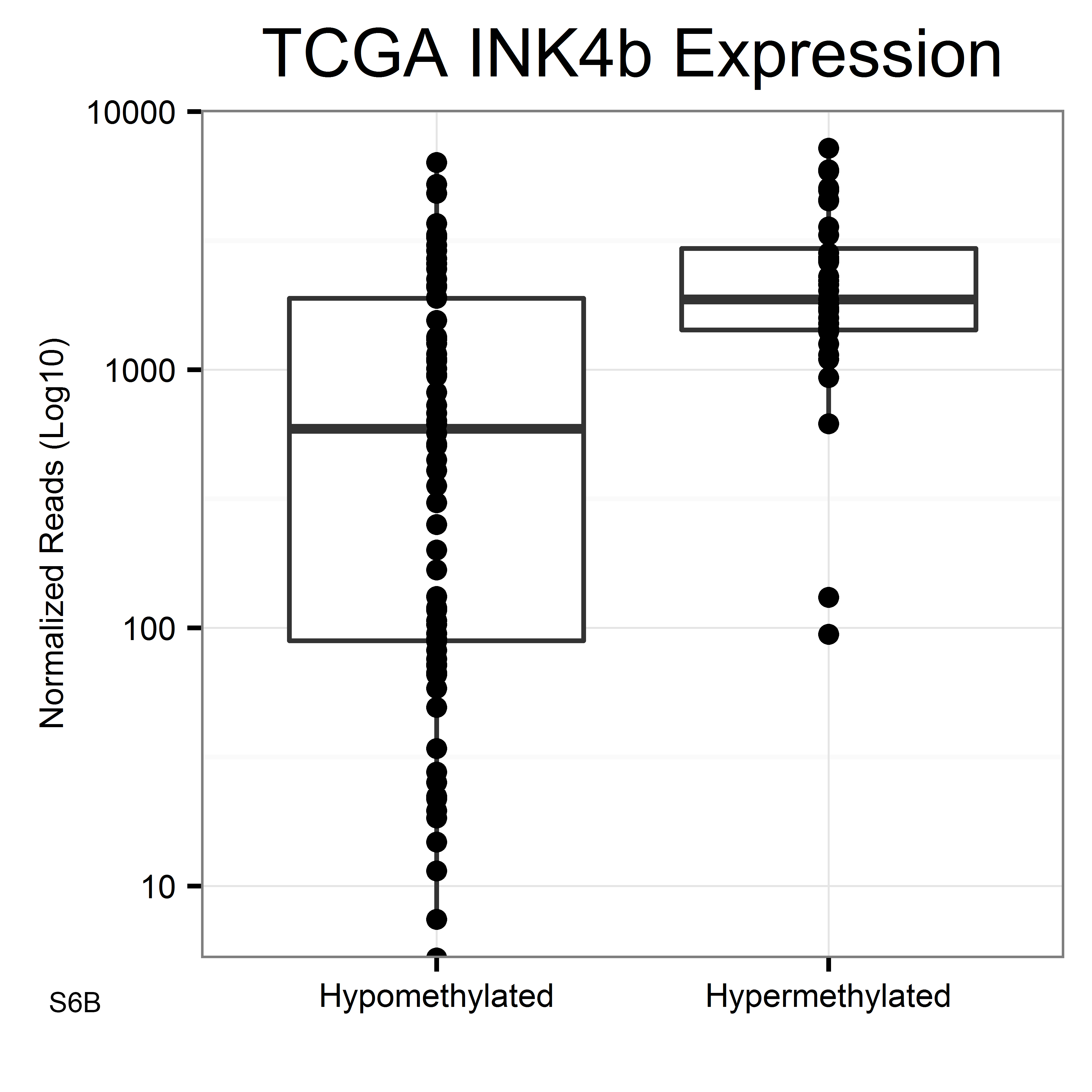

Supplement: Supplementary file 15 [file CAM4-6-397-s015.tif]

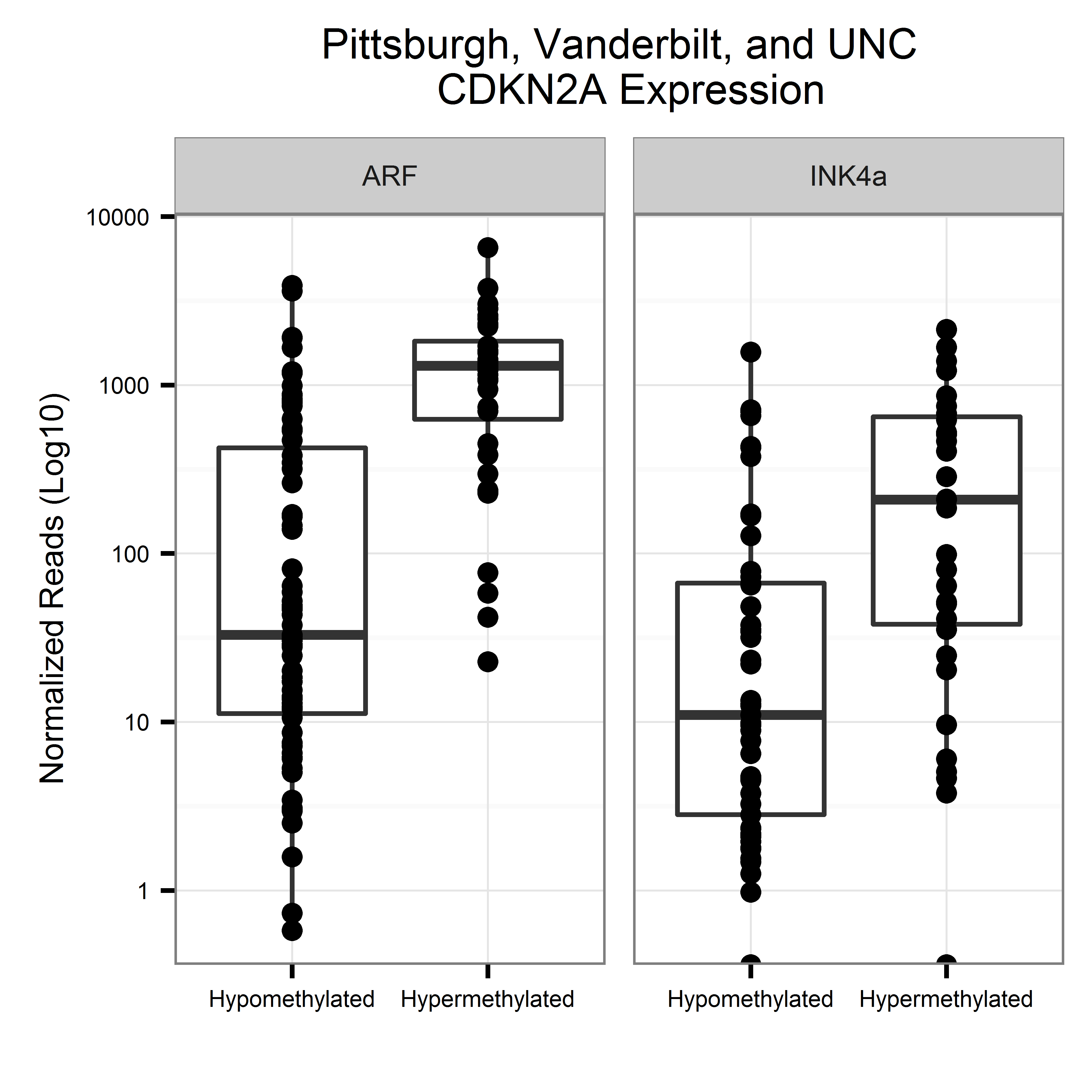

Supplement: Supplementary file 16 [file CAM4-6-397-s016.tif]

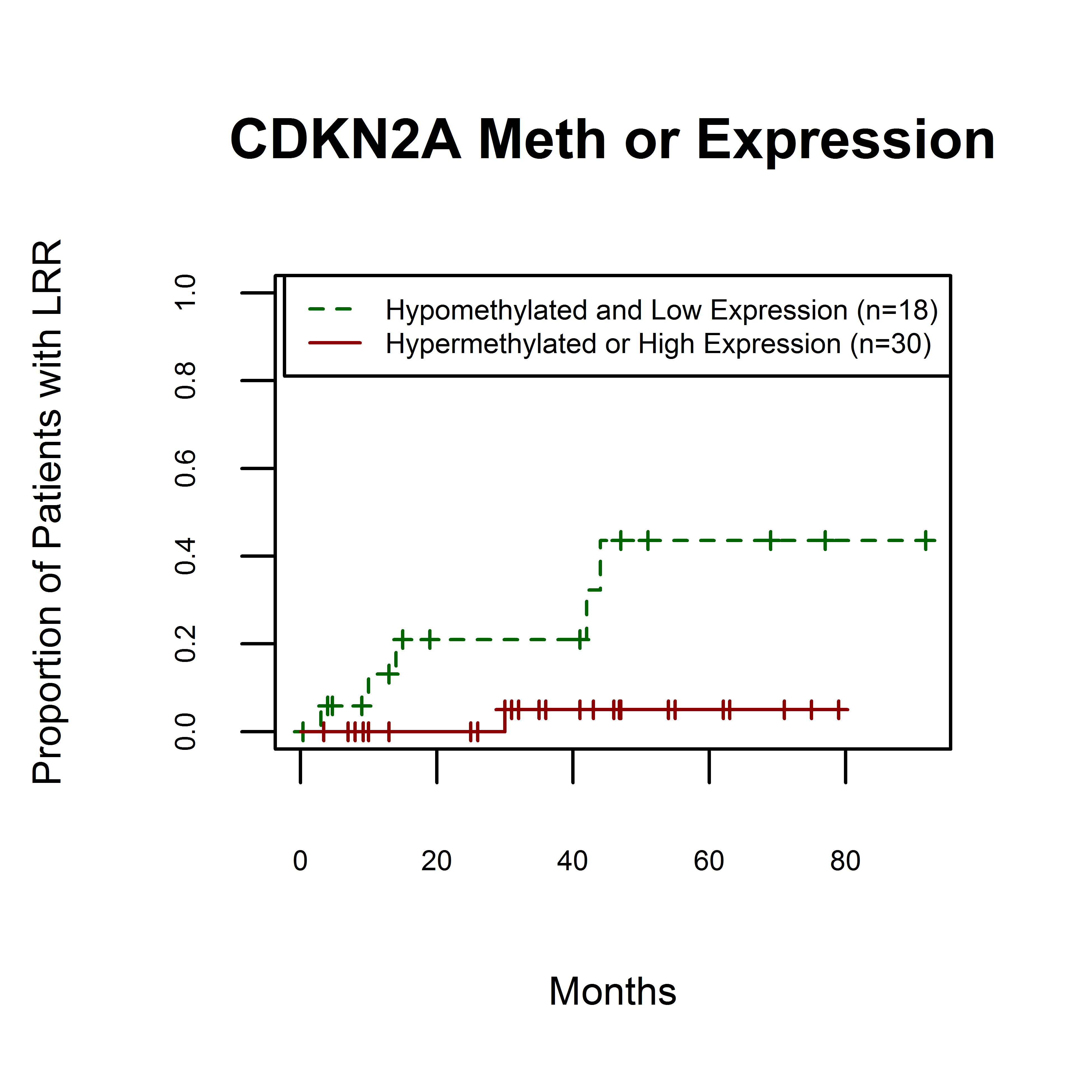

Supplement: Supplementary file 17 [file CAM4-6-397-s017.tif]
