# Supplementary material for: Nonpromoter methylation of the CDKN2A gene with active transcription is associated with improved locoregional control in laryngeal squamous cell carcinoma
Source: Cancer Med. 2017 Jan 19;6(2):397–407. doi: 10.1002/cam4.961 (PMC5313649; doi:10.1002/cam4.961)
Supplement: Supplementary file 18 [file CAM4-6-397-s018.docx]

**Supplemental Table 1*.*** *Clinical Data for the Montefiore Medical Center Cohort*. Percentages taken for the number of patients out of the entire cohort (n=43 samples).

| **Cohort Characteristics** | **Hypomethylated** | **Hypermethylated** | **Fisher's Exact Test** | **Total N** | **%** |
| --- | --- | --- | --- | --- | --- |
| **Age** | | | | | |
| ≤ 60 | 12 | 6 | 1.0 | 18 | **42** |
| > 60 | 17 | 8 |  | 25 | **58** |
| **Race** | | | | | |
| African American | 12 | 4 | 0.5 | 16 | **37** |
| Caucasian | 17 | 10 |  | 27 | **63** |
| **Ethnicity** | | | | | |
| Hispanic/Latino | 4 | 3 | 0.7 | 7 | **16** |
| non-Hispanic/Latino | 25 | 11 |  | 36 | **84** |
| **Gender** | | | | | |
| Male | 18 | 8 | 1.0 | 26 | **60** |
| Female | 11 | 6 |  | 17 | **40** |
| **Clinical Stage** | | | | | |
| I | 2 | 0 | 1.0* | 2 | **5** |
| II | 5 | 3 |  | 8 | **19** |
| III | 10 | 3 |  | 13 | **30** |
| IV | 12 | 8 |  | 20 | **46** |
| **Alcohol Consumption** | | | | | |
| Current | 3 | 4 | 0.2 | 7 | **16** |
| Former/Never | 26 | 9 |  | 35 | **81** |
| **Tobacco Smoking** | | | | | |
| Current | 13 | 4 | 0.3 | 17 | **40** |
| Former/Never | 16 | 10 |  | 26 | **60** |
| **HPV Status** | | | | | |
| RNA Positive | 3 | 2 | 1.0 | 5 | **12** |
| RNA Negative | 18 | 10 |  | 28 | **65** |
| **P16 Staining** |  |  |  |  |  |
| Positive | 5 | 7 | 0.03 | 12 | **28** |
| Negative | 23 | 6 |  | 29 | **67** |

* Fisher’s exact test between stages I-II and stages III-IV

**Supplemental Table 2.** *Clinical Data for the Patients from the Montefiore Medical Center Cohort with qRT-PCR Data*. Percentages taken for the number of patients out of the entire cohort (29 samples).

| **Cohort Characteristics** | **Hypomethylated** | **Hypermethylated** | **Fisher's Exact Test** | **Total N** | **%** |
| --- | --- | --- | --- | --- | --- |
| **Age** | | | | | |
| ≤ 60 | 6 | 6 | 0.4 | 12 | **41** |
| > 60 | 12 | 5 |  | 17 | **59** |
| **Race** | | | | | |
| African American | 9 | 3 | 0.3 | 12 | **41** |
| Caucasian | 9 | 8 |  | 17 | **59** |
| **Ethnicity** | | | | | |
| Hispanic/Latino | 3 | 4 | 0.4 | 7 | **24** |
| non-Hispanic/Latino | 15 | 7 |  | 22 | **76** |
| **Gender** | | | | | |
| Male | 15 | 6 | 0.2 | 21 | **72** |
| Female | 3 | 5 |  | 8 | **28** |
| **Clinical Stage** | | | | | |
| I | 1 | 0 | 1.0* | 1 | **3** |
| II | 3 | 2 |  | 5 | **17** |
| III | 4 | 2 |  | 6 | **21** |
| IV | 10 | 7 |  | 17 | **59** |
| **Alcohol Consumption** | | | | | |
| Current | 2 | 2 | 1.0 | 4 | **14** |
| Ever | 7 | 7 |  | 14 | **48** |
| **Tobacco Smoking** | | | | | |
| Current | 8 | 3 | 0.4 | 11 | **38** |
| Former/Never | 10 | 8 |  | 18 | **62** |
| **HPV Status** | | | | | |
| RNA Positive | 2 | 1 | 1.0 | 3 | **10** |
| RNA Negative | 12 | 9 |  | 21 | **72** |
| **P16 Staining** |  |  |  |  |  |
| Positive | 3 | 6 | 0.03 | 9 | **31** |
| Negative | 15 | 4 |  | 19 | **66** |

* Fisher’s exact test between stages I-II and stages III-IV

**Supplemental Table 3.** *Clinical Data for TCGA laryngeal tumors.* Percentages taken for the number of patients out of the entire cohort (n=111 samples).

| **Cohort Characteristics** | **Hypomethylated** | **Hypermethylated** | **Fisher's Exact Test** | **Total N** | **%** |
| --- | --- | --- | --- | --- | --- |
| **Age** | | | | | |
| ≤ 60 | 34 | 12 | 0.07 | 46 | **41** |
| > 60 | 36 | 29 |  | 65 | **59** |
| **Race** | | | | | |
| African American | 13 | 1 | 0.06* | 14 | **13** |
| Asian | 0 | 1 |  | 1 | **1** |
| American Indian or Alaskan Native | 1 | 0 |  | 1 | **1** |
| Caucasian | 59 | 32 |  | 91 | **82** |
| **Ethnicity** | | | | | |
| Hispanic/Latino | 3 | 2 | 1.0 | 5 | **5** |
| non-Hispanic/Latino | 65 | 33 |  | 98 | **88** |
| **Gender** | | | | | |
| Male | 61 | 31 | 0.6 | 92 | **83** |
| Female | 14 | 5 |  | 19 | **17** |
| **Clinical Stage** | | | | | |
| I | 2 | 0 | 0.5† | 2 | **2** |
| II | 6 | 6 |  | 12 | **11** |
| III | 15 | 12 |  | 27 | **24** |
| IV | 48 | 17 |  | 65 | **59** |
| **Tobacco Smoking** | | | | | |
| Current | 39 | 17 | 0.4 | 56 | **50** |
| Former/Never | 30 | 19 |  | 49 | **44** |
| **HPV Status** | | | | | |
| RNA Positive | 2 | 4 | 0.08 | 6 | **5** |
| RNA Negative | 70 | 30 |  | 100 | **90** |

* Fisher’s exact test between African American and all other races

† Fisher’s exact test between clinical stages I-II and clinical stages III-IV

**Supplemental Table 4.** *Clinical Data for the University of Pittsburgh, Vanderbilt University, and University of North Carolina of Chapel Hill Cohorts.* Percentages taken for the number of patients out of the entire cohort (n=40 samples).

| **Cohort Characteristics** | **Hypomethylated** | **Hypermethylated** | **Fisher’s Exact Test** | **Total N** | **%** |
| --- | --- | --- | --- | --- | --- |
| **Age** | | | | | |
| ≤ 60 | 12 | 5 | 0.3 | 17 | **43** |
| > 60 | 12 | 11 |  | 23 | **57** |
| **Race** | | | | | |
| African American | 7 | 0 | 0.03 | 7 | **18** |
| Caucasian | 17 | 16 |  | 32 | **80** |
| **Ethnicity** | | | | | |
| Hispanic/Latino | 0 | 0 | 1.0 | 0 | **0** |
| non-Hispanic/Latino | 24 | 16 |  | 40 | **100** |
| **Gender** | | | | | |
| Male | 6 | 5 | 0.7 | 11 | **28** |
| Female | 18 | 11 |  | 29 | **72** |
| **Clinical Stage** | | | | | |
| I | 0 | 0 | 0.3* | 0 | **0** |
| II | 1 | 3 |  | 4 | **10** |
| III | 7 | 4 |  | 11 | **28** |
| IV | 16 | 9 |  | 25 | **62** |
| **Tobacco Smoking** | | | | | |
| Current | 18 | 11 | 0.7 | 29 | **72** |
| Ever | 6 | 5 |  | 11 | **28** |
| **HPV Status** | | | | | |
| RNA Positive | 0 | 0 | 1.0 | 0 | **0** |
| RNA Negative | 20 | 10 |  | 30 | **75** |

* Fisher’s exact test between clinical stages I-II and clinical stages III-IV
