# Supplementary material for: Nonpromoter methylation of the CDKN2A gene with active transcription is associated with improved locoregional control in laryngeal squamous cell carcinoma
Source: Cancer Med. 2017 Jan 19;6(2):397–407. doi: 10.1002/cam4.961 (PMC5313649; doi:10.1002/cam4.961)
Supplement: Supplementary file 19 [file CAM4-6-397-s019.docx]

**Supplemental Figure 1.** ARF and all CDKN2A variants are overexpressed in laryngeal tumors with downstream CDKN2A methylation. To validate the qRT-PCR results, 33 LXSCC were assayed for ARF and total CDKN2A expression by microarray. Relative mRNA expression levels for 21 hypomethylated tumors were compared to 12 hypermethylated tumors and plotted on a Log10 scale. Increased levels of ARF (Wilcoxon rank-sum, p<0.0001) and total CDKN2A (p<0.0001) expression was observed for the hypermethylated groups.

**Supplemental Figure 2.** *ARF expression is associated with CDKN2A downstream methylation.* Relative ARF expression levels were plotted against M-values for each CpG assayed in the downstream region of the CDKN2A locus. Methylation cutoffs for each individual CpG were selected by applying a percentile threshold of 32% determined by cluster analysis (Figure 1), and is represented by the blue vertical line. Black circles represent tumors that were designated as hypermethylated by hierarchical clustering. The red horizontal line represents the cutoff for ARF expression used for outcome analysis. **S1A.** ARF expression was plotted against the M-values for cg07752420. All of the hypermethylated tumors were identified using this threshold. **S1B.** ARF expression was plotted against the M-values for cg09099744. All of the hypermethylated tumors were identified using this threshold. **S1C.** ARF expression was plotted against the M-values for cg10895543. Using this threshold, 82% of the hypermethylated tumors were identified. **S1D.** ARF expression was plotted against M-values for cg11653709. Using this threshold, 91% of the hypermethylated tumors were identified.

**Supplemental Figure 3.** *INK4a expression is associated with non-promtoer CDKN2A methylation.* Relative INK4a expression levels were plotted against M-values for each CpG assayed in the downstream non-promoter region of CDKN2A. Methylation cutoffs for each individual CpG were selected by applying a percentile threshold of 32% determined by cluster analysis (Figure 1), and are represented by a blue vertical line. Black circles represent tumors that were designated as hypermethylated by hierarchical clustering. The red horizontal line represents the cutoff for INK4a expression used for outcome analysis. **Figure** **S2A:** INK4a expression plotted against the M-values for cg07752420. All of the hypermethylated tumors were identified using this threshold. **S2B:** INK4a expression plotted against the M-values for cg09099744. All of the hypermethylated tumors were identified using this threshold. **S2C:** INK4a expression plotted against the M-values for cg10895543. Using this threshold, 82% of the hypermethylated tumors were identified. **S2D:** INK4a expression plotted against M-values for cg11653709. Using this threshold, 91% of the hypermethylated tumors were identified.

**Supplemental Figure 4**. *ARF and INK4a expression are associated with CDKN2A non-promoter methylation in TCGA laryngeal tumors*. Relative ARF and INK4a expression levels were plotted against M-values for the CpG assayed in the downstream non-promoter region of CDKN2A assayed in TCGA LXSCC samples (n=111). The blue vertical line represents the methylation cutoff used for TCGA samples. The red horizontal line represents the cutoff for ARF and INK4a expression based on median expression values (171 and 35 RPKM, respectively). **Figure** **S4A:** ARF expression plotted against the M-values for cg12840719. **S4B:** INK4a expression plotted against the M-values for cg12840719.

**Supplemental Figure 5.** *Expression of Cyclin A and Cyclin E are similar between hypomethylated and hypermethylated laryngeal tumors in TCGA.* **S5A.** RNA expression levels for the Cyclin A transcript were obtained by RNAseq, and compared for 75 hypomethylated vs.36 hypermethylated tumors. There was no significant difference between the two groups (Wilcoxon rank-sum, p=0.8). **S5B.** RNA expression levels for the Cyclin A transcript were obtained by RNAseq, and compared for 75 hypomethylated vs.36 hypermethylated tumors. There was no significant difference between the two groups (Wilcoxon rank-sum, p=0.2).

**Supplemental Figure 6.** *INK4b expression is increased in hypermethylated laryngeal tumors from MMC and TCGA Cohorts.* **S6A.** RNA levels for the INK4b transcript were obtained by microarray, and compared for 21 hypomethylated vs. 12 hypermethylated LXSCC tumors. There was a significant increase in INK4b expression between the two groups (Wilcoxon rank-sum, p=0.015). **S6B.** Expression for the INK4b transcript were obtained from TCGA RNAseq data. RNA levels for 75 hypomethylated laryngeal tumors were compared to 36 hypermethylated tumors, and plotted on a Log10 scale. There was a significant increase in INK4b expression in hypermethylated tumors when compared to hypomethylted tumors (Wilcoxon rank-sum, p<0.0001).

**Supplemental Figure 7.** *ARF and INK4a expression are increased in hypermethylated laryngeal tumors from TCGA.* RNAseq expression values for ARF and INK4a were plotted for 40 primary LXSCC tumors from the University of Pittsburgh, Vanderbilt University, and University of North Carolina cohorts included in our clinical validation analyses. Expression levels were significantly higher in the 24 hypomethylated vs. 16 hypermethylated LXSCC tumors for both ARF (Wilcoxon rank-sum, p < 0.001) and INK4a (Wilcoxon rank-sum, p=0.005) splice variants.

**Supplemental Figure 8.** *Non-promoter CDKN2A hypomethylation and low ARF/INK4a expression in tumors is associated with an increased risk of local regional recurrence in laryngeal cancer patients treated with surgery.* Time to loco-regional recurrence was assessed for 48 patients that were treated with surgery as the primary modality across the MMC, Pittsburgh, Vanderbilt, and UNC cohorts and compared with respect to non-promoter CDKN2A methylation and ARF/INK4a RNA expression status using previously established cut-offs. A Kaplan-Meier plot was generated for 18 hypomethylated tumors with low levels of CDKN2A expression (green, dashed line) and 30 tumors with either hypermethylation or high levels of CDKN2A expression (red, solid line). The results indicate a significantly lower risk of LRR in patients with CDKN2A hypermethylation, high ARF expression, or high INK4a expression (Log-rank, p=0.01).
